# Green exfoliation of 2D nanomaterials using cyrene as a solvent

**DOI:** 10.1039/d5na00576k

**Published:** 2025-10-14

**Authors:** Pedro Moreira, João Mendes, Tomás Calmeiro, Daniela Nunes, David Carvalho, Adam Kelly, Hugo Águas, Elvira Fortunato, Rodrigo Martins, Joana Vaz Pinto, João Coelho, Emanuel Carlos

**Affiliations:** a CENIMAT|i3N, Department of Materials Science, School of Science and Technology, NOVA University Lisbon and CEMOP/UNINOVA Campus de Caparica 2829-516 Caparica Portugal e.carlos@fct.unl.pt; b Department of Condensed Matter Physics, Institute of Materials Science of Seville, University of Seville—CSIC Avenida Reina Mercedes SN 41012 Seville Spain jmesquita@us.es

## Abstract

Liquid-phase exfoliation (LPE) is a versatile and scalable method for producing high-quality two-dimensional materials (2DMs). However, commonly used solvents such as dimethylformamide (DMF) or *N*-methyl-2-pyrrolidone (NMP) are highly toxic, limiting their potential for large-scale industrial applications. In this study, we address this challenge using Cyrene (dihydrolevoglucosenone), a nontoxic and biodegradable solvent, for the exfoliation of several materials, including graphene, MoS_2_, WS_2_, MoO_3_, V_2_O_5_, and hBN (hexagonal boron nitride). Exfoliation was carried out using low-powered bath sonication, a cost effective and energy efficient method and optimization was conducted to maximize the final concentration of exfoliated material. To assess the potential of Cyrene for LPE, extensive characterization and comparison of the produced 2DMs with their precursors was performed. The highest ink concentrations were observed for MoS_2_ (2.6 mg mL^−1^), followed by hBN (2.3 mg mL^−1^) and V_2_O_5_ (1.9 mg mL^−1^), demonstrating the ability of Cyrene to effectively stabilize a variety of 2D materials in dispersion. Structural and morphological properties of the exfoliated materials were characterized using X-ray diffraction (XRD), Raman spectroscopy, UV-vis spectroscopy, scanning electron microscopy (SEM) and high-angle annular dark-field scanning transmission electron microscopy (HAADF-STEM). XRD patterns mainly showed only one reflection revealing the oriented nature of the materials, with significant broadening of the full width at half maximum (FWHM) compared to the original materials. Also, Raman spectroscopy spectra for graphene showed ratios characteristic of multi-layered structures and SEM imaging revealed a broad distribution of flake sizes. This work highlights the potential of Cyrene as a sustainable and efficient solvent for LPE of diverse 2D materials. The systematic optimization method presented here achieves high dispersion concentrations in a repeatable manner using low-power and ecofriendly means. These findings establish a foundation for the scalable production of 2D inks, enabling their use in advanced applications such as electrode, dielectric and semiconductor layers of electronic devices.

## Abstract 2

Liquid phase exfoliation in green solvent Cyrene combines favorable solubility properties with low toxicity, yielding stable dispersions of diverse 2D materials. This sustainable approach highlights Cyrene as a viable alternative to NMP and DMF.

## Graphical abstract

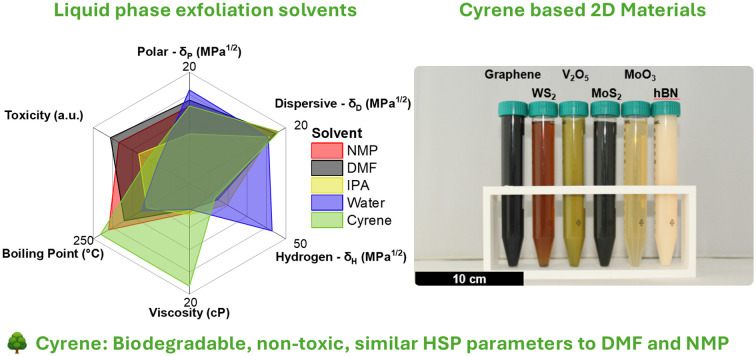

## 1. Introduction

In 1994, graphene was first defined by Boehm, *et al.*^1^ as a one-atom-thick carbon layer. Ten years later, in 2004, Novoselov, *et al.*^2^ introduced a simple method to produce monolayer (MLG) and few-layer graphene (FLG) by systematically peeling carbon films from highly oriented pyrolytic graphite.^3^ Three decades after its introduction, graphene is being researched for its unique properties in energy storage,^4,5^ solar cells,^6,7^ printed electronics,^8,9^ water treatment,^10^ and biomedical applications.^10,11^ The introduction of reliable methods of production for graphene has sparked interest in other 2D materials.^3,5^

Transition metal dichalcogenides (TMDs) belong to the family of 2DMs and are described with the general formula of MX_2_, where M stands for a transition metal and X is a chalcogen.^12^ Unlike graphene, many monolayers of these TMDs are natural semiconductors, showcasing bandgaps of 1–2 eV (ref. 13–15) making them suitable for application in, thin-film transistors (TFTs),^12,16,17^ photodetectors,^6,16^ and photodiodes.^7^ One of such materials is molybdenum disulfide (MoS_2_), first prepared in 1989 by Gutiérrez and Henglein^18^ using liquid-phase exfoliation^9^ and recently, in 2023, the same technique has been utilized by Adam, *et al.*^11^ to synthesize MoS_2_ and tungsten disulfide (WS_2_). In fact, LPE, has been recognized as a reliable and straightforward way of obtaining large quantities of 2DMs with reasonably good quality and large surface areas.^3,6,9–11,19^ During LPE, a solvent is used as medium to disperse a material in its bulk form and energy is provided to the system, usually by probe sonication, promoting the delamination of the layered crystals into thin nanosheets.^5^ The stability of dispersions is ensured by the compatibility between exfoliated layers and the solvent which can be described by the Hansen solubility parameters (HSP) of the solvent and material. These are a set of three numerical values describing different intermolecular forces: polar interactions (*δ*_P_), arising from dipole–dipole interactions in polar solvents; dispersive interactions (*δ*_D_), from weak van der Waals and London dispersion forces; and hydrogen bonding (*δ*_H_) coming from hydrogen donor/accepter interactions in alcohols and water. Plotting a material's Hansen solubility parameters (HSP) in a 3D space, along with a solubility radius based on known compatible solvents, provides valuable insights into other potential solvents that may ensure its stability.^9,20–22^ Additionally, several studies emphasize the importance of solvent surface energy, viscosity, temperature and sonication method in controlling yield.^23,24^ However, the surface energy of the solvent is considered a crude approach to exfoliation. In contrast, the HSP theory is considered the key to maximizing exfoliation while controlling other parameters.^25^ Based on these parameters and experimental results, NMP and DMF have emerged as the most suitable options in LPE of 2DMs with some impressive results published, including exfoliation of graphene, MoS_2_, and WS_2_ with final concentrations up to 2 mg mL^−1^.^20,26–29^

However, the toxic nature of both solvents hinders their scale up, and as such, several approaches have been developed in mixtures of water and surfactants. For instance, Paton *et al.*, based on a shear mixing method, exfoliated a series of layered crystals in aqueous surfactant solutions (sodium cholate, NaCh).^30^ However, the presence of the surfactant may pose problems in the manufacture of a device, as it may introduce contaminants or require additional processing steps to remove residues.^31^ In this context, Cyrene is an emerging green solvent with potential for use in the exfoliation of graphene due to its unique physicochemical properties. These capabilities can be explained by its solubility parameters (*δ*_P_ = 10.8 MPa^1/2^, *δ*_D_ = 18.7 MPa^1/2^ and *δ*_H_ = 6.9 MPa^1/2^), which are quite similar to those measured for NMP (*δ*_P_ = 9.3 MPa^1/2^, *δ*_D_ = 18.0 MPa^1/2^ and *δ*_H_ = 7.7 MPa^1/2^) and graphite reference values (*δ*_P_ = 12.3 MPa^1/2^, *δ*_D_ = 18.0 MPa^1/2^ and *δ*_H_ = 7.2 MPa^1/2^).^20,31,32^ Interestingly, studies show that Cyrene can efficiently exfoliate graphene through LPE.^9,20,29,31,33^ In 2017, Salavagione, *et al.*^31^ showed Cyrene-processed graphene with concentrations as high as 0.24 mg mL^−1^, which is an order of magnitude larger than the concentration of 0.018 mg mL^−1^ observed for NMP under the same processing conditions. Tkachev, *et al.*^29^ utilized a combination of tip-sonication and shear mixing to achieve a concentration of 3.70 mg mL^−1^ of few-layer graphene in Cyrene. In contrast, NMP and DMF only achieved concentrations of 1.61 mg mL^−1^ and 0.30 mg mL^−1^, respectively. Finally, in 2018, Pan, *et al.*^33^ reported a concentration of 10 mg mL^−1^ using sonication-assisted exfoliation.

Besides its exfoliating potential, Cyrene is derived from renewable biomass sources, such as cellulose. It's non-toxic, biodegradable properties make it an attractive alternative to NMP or DMF in graphene production. The green credentials of Cyrene make it an ideal candidate for scaling up graphene production without causing environmental harm, aligning with sustainability goals in materials science and industrial processes. In this work we investigate the capabilities of Cyrene for the exfoliation of several 2DMs including graphene, hBN, TMDs (MoS_2_ and WS_2_) and TMOs (MoO_3_ and V_2_O_5_). We found competitively high concentrations for most materials, in line with reported values of LPE values in typical toxic solvents, of mostly multi-layer content using low-power sonication techniques. Minimal amounts of defects or deformation of the crystalline structures of the precursor powders were observed by Raman spectroscopy, XRD results, and high-resolution transmission electron spectroscopy (HRTEM) imaging. In fact, the authors have recently demonstrated that graphene dispersed in Cyrene is a suitable material for the fabrication of sustainable supercapacitors.^4^ The easy, cost-effective, and optimized sustainable production of these materials paves the way for their implementation in the design of different devices. For instance, they can be implemented in different applications, including as semiconductor or sensing layers (MoS_2_ and WS_2_) in thin-film transistors;^34–37^ as electrodes in various devices,^38–41^ including supercapacitors and batteries (V_2_O_5_, MoO_3_, hBN-WS_2_);^37,42,43^ as anti-oxidation coatings (hBN);^38,44^ as deep UV-emitting devices; photodetectors (MoS_2_)^35^ and as a dielectric (hBN).^43–45^ For this reason, the exfoliation of these nanomaterials in Cyrene is a crucial option for the future of sustainable printed electronics.

## 2. Experimental section

### 2.1 Ink preparation

2DM dispersions were prepared in 30 mL of Cyrene (CAS: 53716-82-8, from Sigma-Aldrich), from the following precursors: graphite (CAS: 7782-42-5 from Sigma-Aldrich), MoS_2_ (1317-33-5 from Sigma-Aldrich), V_2_O_5_ (CAS: 1314-62-1 from Sigma-Aldrich), WS_2_ (CAS: 12138-09-9 from Sigma-Aldrich), MoO_3_ (CAS: 1313-27-5 from Sigma-Aldrich) and hBN (CAS: 10043-11-5 from Sigma-Aldrich). Then, LPE of each material was done in a Fisherbrand FB11207 ultrasound bath sonicator, rated for a maximum output of 330 W at a frequency of 37 kHz and 100% power. Optimization of the ink's final concentration (*C*_f_) was performed in a two-step process. First the initial concentration (*C*_i_) of precursor material was varied and dispersions were prepared with 5, 10, 30, 50, 70, 100, 150 and 200 mg mL^−1^ and then exfoliated for 8 h. The stable setting with the highest final concentration was selected and further optimized by adjusting the ultrasound bath duration: 2, 4, 6, 8, 10, 12, 14, and 18 h. During all exfoliations, the bath water was refreshed hourly to prevent overheating, ensuring the temperature remained below 45 °C at any given moment.

Afterwards all vials were centrifuged at 500 rpm for 30 min and the supernatant was collected to be again centrifuged at 6000 rpm for 90 minutes (Neya 8 bench top centrifuge) for determination of concentration. The supernatant was carefully pipetted into new vials, and the sedimented material was discarded. Each sample was then filtered through 20 nm filters (0.02 μm, 47 mm Anodisc™ 47 from Cytiva) by pipetting 2–10 mL of solution and rinsing with copious amounts of IPA to remove excess Cyrene. To facilitate the filtration, the ink was initially pipetted into a container with a large amount of 2-propanol (IPA), stirred until mixing was complete and only then poured into the filtration rig. Then, all samples were dried at 40 °C overnight in vacuum to ensure any leftover IPA was evaporated followed by the determination of their final concentration from the weights of the filters before and after filtration. The yield (eqn (1)) of each sample was then determined as a percentage of the ratio between the final ink concentration after centrifugation (*C*_f_) and initial precursor concentration (*C*_i_).E1
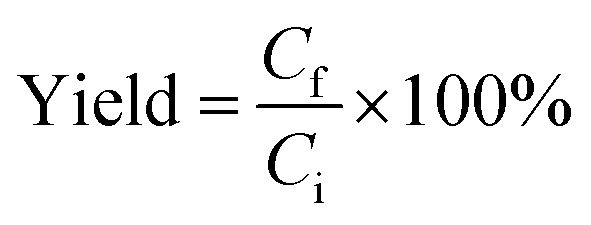


Inks with optimized settings (*C*_i_ and ultrasound time) were prepared anew, centrifuged at 500 rpm for 30 min and the supernatant collected. On these samples a centrifugation cascade was performed (1500, 3000, 4500 and 6000 rpm) and the supernatant of each step was collected for characterization.

### 2.2 Ink characterization

Optimized inks were successively diluted by taking a known volume and adding the same amount of Cyrene from 1 : 0 (ink : Cyrene) to as low as 1 : 63 and characterized by UV-vis spectroscopy (PerkinElmer UV/VIS/NIR Spectrometer Lambda 365+) in a range of *λ* = 200 to 1400 nm with a 480 nm min^−1^ scan rate and a step of 1 nm and plotted in terms of extinction per unit length. The absorbance values of photons with an energy of 1.88 eV (*λ* = 660 nm) were taken from non-saturated measurements for all materials except MoS_2_ for which the values taken were at an energy of 2.1 eV (*λ* = 590 nm). According to the Lambert–Beer law (eqn (2)), the extinction coefficient (*∈*) was determined for each dilution from the extinction (*E*), the light's linear path length trough the cuvette (*L*), and the concentration (*c*), such that^46^

E2 *E* = *∈cL*

These values were plotted to extract the specific extinction coefficient of each material ink. We note that Cyrene strongly absorbs radiation below *λ* = 400 nm, therefore the calibration was performed at higher wavelength as to be a useful concentration estimation tool in future batches.

Both the precursor powders and the filtered and dried materials were analyzed in XRD (Aeris from PANalytical in a range of 2*θ* = 10 to 90° over a 15 minutes runtime measurement using a Cu κ_α_ anode with *λ* of 1.54 Å). The crystalline size (*D*) was estimated by Scherrer's equation (eqn (3))E3
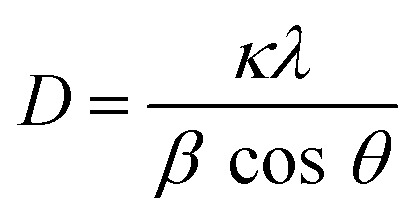
Here, *λ* refers to the beam wavelength (*λ* = 1.54 Å), *κ* is the Scherrer constant with a value of 0.98, *β* is the full width at half maximum (FWHM) of the most intense peak for each sample, and *θ* is the Bragg angle of the peak. An estimation of the interlayer spacing (*d*) was also calculated from Bragg's law (eqn (4)), whereE4
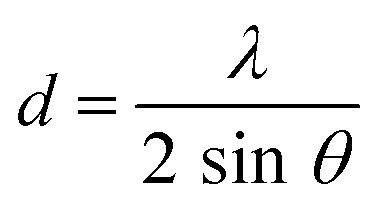


Micro-Raman spectroscopy (Reinshaw inVia Raman microscope) was done to evaluate the quality of exfoliation of each 2DM in a Renishaw inVia Qontor confocal Raman Microscope by focusing either a 532 nm frequency doubled Nd:YAG DPSS excitation laser (Renishaw RL532C50) or a 633 nm HeNe Laser (Renishaw RL633) at varying power values, on the samples using a Leica Nplan 50× objective (NA 0.75, WD 0.37 mm) or a Leica Nplan 100× objective (NA 0.85) to achieve laser spots with sizes between 0.8 and 1.0 μm^2^. An 1800 l mm^−1^ grating was used in all cases with exposure time varied from 2 to 10 seconds and a total of 10 accumulations for all materials. A table with the spectra acquisition conditions is available in the SI (Table S4). SEM imaging (Regulus 8220 Scanning Electron Microscope, Hitachi) was used to compare the exfoliated nanosheets with the precursor powders at amplifications ranging from 10 k to 100 k with a beam energy of 5 keV and a current of 10 μA, adjusted as needed. On samples with low conductivity, a 20 nm gold/palladium coating was applied using a Quorum Q150T ES.

AFM samples were prepared by centrifuging 10 mL of each optimized ink at 6000 rpm for two additional hours. The supernatant was discarded, and the deposited material was redispersed in 30 mL of IPA using 10 minutes of ultrasound bath. The resulting redispersions were then centrifuged again at a rate of 6000 rpm for two h, the supernatant was discarded while the deposited material was once again redispersed in a similar manner. Then all samples were systematically diluted in IPA until a low optical density was observed. Si substrates were prepared with an immersion in KOH 1 M solution and placed in an ultrasonic bath for two minutes, followed by being rinsed in ultra-pure (UP) water twice and dried under N_2_ jetting gun. Samples were slowly dropcast onto the Si substrates, which were heated to 120 °C so that the Leidenfrost effect was observed and until regions of the material became visible on the substrate. AFM images were acquired to measure the thickness of platelets on a Park Systems FX40 operated in ambient room conditions in oscillatory mode, using commercially available silicon probes (PPP-NCHR, *f*_0_ = 320 kHz, *r* = < 7 nm; Nanosensors, Switzerland). Images were subjected to low-level order flattening where required. In Scanning transmission electron microscopy (STEM) analysis with High-Angle Annular Dark-Field (HAADF) imaging, inks were similarly prepared into dilutions of low optical density, pipetted onto TEM grids and allowed to rest in air at room temperature until all solvent was evaporated. A Hitachi HF5000 probe-corrected field-emission transmission electron microscope was used, operating at 200 kV.

## 3. Results and discussion

**Fig. 1:**
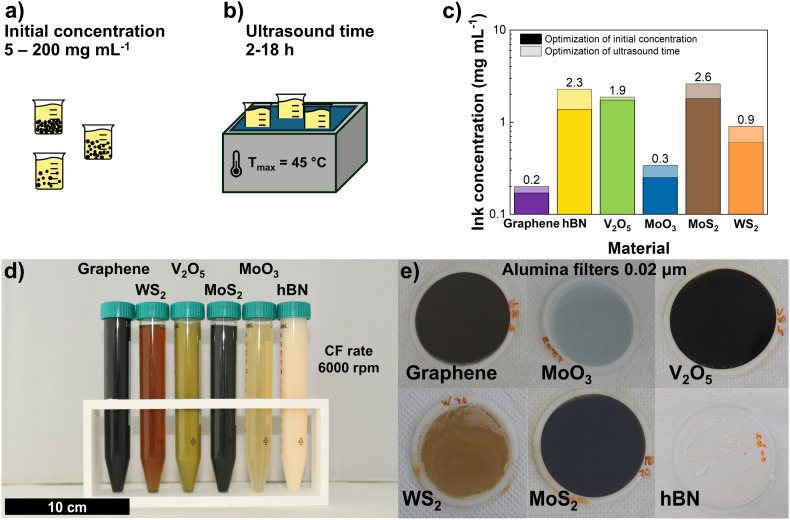
Optimization of final concentration by varying: (a) initial concentration (5–200 mg mL^−1^) and (b) ultrasound time (2–18 h). (c) Highest concentrations obtained in each series for all materials. (d) Optimized inks and (e) filtered inks in 0.02 μm alumina filters.

In this section we present the characterization of the exfoliated materials and inks, separated into three groups: graphene and hBN, TMDs (WS_2_ and MoS_2_) and TMOs (V_2_O_5_ and MoO_3_). The exfoliation process was optimized to maximize ink concentration by first varying the initial concentration for a fixed sonication time of 10 h and CF rate of 6000 rpm (Fig. 1(a)). The stable suspensions with the highest *C*_f_ were found to be 0.17 mg mL^−1^ for graphene (*C*_i_ = 50 mg mL^−1^), 1.37 mg mL^−1^ for hBN (*C*_i_ = 50 mg mL^−1^), 1.73 mg mL^−1^ for V_2_O_5_ (*C*_i_ = 70 mg mL^−1^), 0.25 mg mL^−1^ for MoO_3_ (*C*_i_ = 50 mg mL^−1^), 1.9 mg mL^−1^ for MoS_2_ (*C*_i_ = 50 mg mL^−1^) and 0.6 mg mL^−1^ for WS_2_ (*C*_i_ = 30 mg mL^−1^). For all materials a minimum *C*_i_ of 30 mg mL^−1^ was required for meaningful exfoliation, stabilizing at either 50 or 70 mg mL^−1^. Next, we took the optimized starting concentration and varied the ultrasound time, keeping the same CF rate (Fig. 1(b)). For all materials, the final concentration increased with sonication time but reached a plateau after which only small increases were observed (Fig. S1). These plateaus occurred for a *C*_f_ of 0.20 mg mL^−1^ for graphene (10 h), 2.27 mg mL^−1^ for hBN (8 h), 1.87 mg mL^−1^ for V_2_O_5_ (8 h), 0.34 mg mL^−1^ for MoO_3_ (10 h), 2.60 mg mL^−1^ for MoS_2_ (10 h) and 0.90 mg mL^−1^ for WS_2_ (14 h). The highest concentrations obtained for each ink in both optimization series are shown in Fig. 1(c). The filtered powders from these inks (Fig. 1(d) and (e)) were analyzed under Raman spectroscopy, XRD, SEM and HRTEM to access crystallinity, number of layers and a comparison with precursor materials was made to check for damage during processing. A comprehensive review regarding concentration, solvents, exfoliation method and layer content of several 2DMs has been compiled in Table S2.

### 3.1 Graphene and hBN

**Fig. 2:**
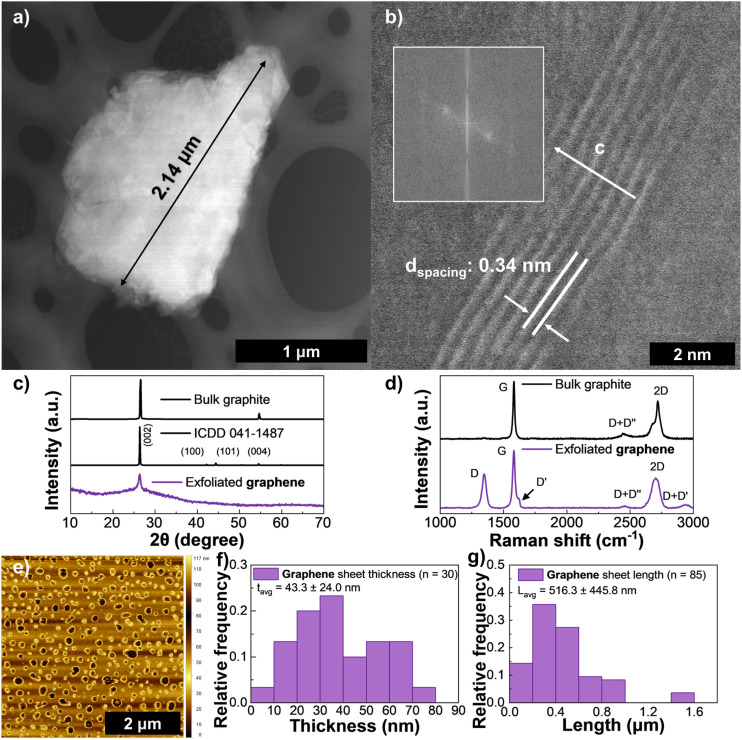
HAADF-STEM of (a) a large, exfoliated graphene sheet and (b) a seven layer stack along the *c* direction (the inset show the power FFT patterns from the HAADF-STEM image). (c) XRD spectra for graphene (bottom), reference hexagonal carbon (bottom) pattern ICDD 041-1487 and bulk graphite flakes (top), (d) Raman spectroscopy of exfoliated graphene (bottom) and bulk graphite flakes (top) and (e) AFM scan of drop-casted ink diluted in IPA. Distribution of graphene sheet thickness *via* AFM (f) and length *via* SEM (g), the error in these reflects one standard deviation.

Graphene produced the lowest concentration out of all the materials with a maximum of 0.2 mg mL^−1^, representing a yield of 0.4% and sedimentation was observed after one week of storage in the dark at room temperature. In Fig. S2(a) the influence of concentration initial concentration is visible by the slight coloration for very low *C*_i_ (below 10 mg mL^−1^) or very high *C*_i_ (above 70 mg mL^−1^) signifying a small *C*_f_. Although lower-power sonication causes less damage to the samples and produces larger flakes, it usually results in lower dispersion concentrations. However, it has been shown that these concentrations can be increased by using tip sonication and/or shear mixing.^29,31^ Güler and Sönmez^23^ have also demonstrated that the use of expanded graphite in solvents such as NMP and DMF provide higher efficiency in surfactant-assisted media. While the utilization of expanded graphite would likely lead to greater outputs, it is also beyond the scope of this study as its production heavily relies on strong acids which do not meet the sustainability objectives set for this study. Fig. 2(a) shows a large flake with a length of 2.14 μm and an interlayer spacing of 3.4 Å was also estimated from a cross-section using HRTEM as depicted in Fig. 2(b), similar to its theoretical value of 3.35 Å and attributed to the spacing of (002) planes along the *c* direction.^19^ The XRD of filtered graphene (bottom spectrum in Fig. 2(c)) shows a peak at 2*θ* = 27° and a less intense feature at 54° (only present in samples prepared at lower rpms, available in Fig. S9(a)), both decreasing in intensity as less material is present at higher centrifugation speeds. These reflections are attributed to the (002) and (004) planes of graphite, as seen in the reference pattern ICDD 041-1487 and the broadening of these features also suggests smaller crystallite sizes for higher CF rates. An initial broad feature is attributed to the filter used to capture the material as it is also present in the blank filter XRD plot (Fig. S9(b)). Our findings align with pattern ICDD 48–1487 as hexagonal carbon/graphene like structures and from the full width at height maximum (FWHM) and Braggs law, the crystallite size was calculated to be 3 nm. Additional XRD results are available in Table S3. The bottom spectrum in Fig. 2(d) contains the Raman spectra of the produced graphene and shows the strongest and most widely studied bands for carbon materials: D (1347 cm^−1^), G (1582 cm^−1^), and 2D (2705 cm^−1^).^10,28,47^ The G band is a result of stretching in sp^2^-hybridized C–C bonds. However, the D feature comes from the edges of nanosheets and defects associated with breathing modes in sp^2^ atoms in hexagonal rings.^25,28^ Bands D′ at 1620 cm^−1^, D + D′′ at 2457 cm^−1^, and D + D′ band at 2942 cm^−1^ are all associated with defects.^47^ The 2D band is simply the second order resonance of the D band.^47^ From the ratio between the 2D and G bands of the graphene sample, the estimated number of layers is calculated to be above 5 (*I*_2D_/*I*_G_ = 0.52).^40,41,48^ The graphite flakes used to prepare the inks (top spectrum) show a sharp G band at 1582 cm^−1^, a blue shifted 2D band centered at 2720 cm^−1^ with a large redshifted shoulder, and a D + D′′ band at 2441 cm^−1^. The shape of the 2D band was analyzed in detail (see Fig. S10) and allows for the clear association of the spectrum of the graphene flakes to that of bulk graphite.^47^ No D band is found in this spectrum, which is expected for graphite.^47^ From AFM (Fig. 2(e)) and SEM imaging (Fig. S2(f)) an average thickness of 43 nm (*n* = 30) and average length of 516 nm (*n* = 85) were extracted as shown in Fig. 2(f) and (g), respectively. As highlighted by Kelly *et al.*,^49^ the aspect ratio of graphene flakes strongly influences intersheet junction resistance, with aspect ratios below ∼50 generally resulting in poorer contact between nanosheets. This can be advantageous in applications such as supercapacitors^4^ or batteries, where increased porosity improves ionic accessibility throughout the electrode network. Additional AFM and SEM imaging for LPE graphene and the remaining materials is available in SI Fig. S16–S18.

An extinction coefficient of 15.13 mg^−1^ mL cm^−1^ was obtained from the measured extinction at *λ* = 660 nm *versus* known concentration, shown in Fig. S2(d), which is within the wide range of reported values for *∈* of solution processed graphene.^31,50^

Hexagonal boron nitride features a structure analogous to graphite, with alternating boron and nitrogen atoms substituting the carbon atoms and a similar interplanar spacing of 3.34 Å (Fig. S3(e)).^32,51,52^ However, unlike graphene, hBN is characterized by a wide bandgap and partially ionic B–N bonds, which gives it unique electronic and chemical properties with applications in lubrication, cosmetics and as a dielectric in electronic devices.^43,51,53,54^ hBN shares with graphene a similar compatibility with various synthesis techniques, including direct growth, mechanical and liquid-phase exfoliation, sputtering, pulsed laser deposition, and chemical vapor deposition, among others.^53,55,56^ We observed a maximum yield of 4.54%, corresponding to an ink with 2.27 mg mL^−1^ and sedimentation starting to occur after 48 h. All inks had a pale-yellow tinted color, becoming increasingly more opaque as the final concentration increased (Fig. S3(a)).

**Fig. 3:**
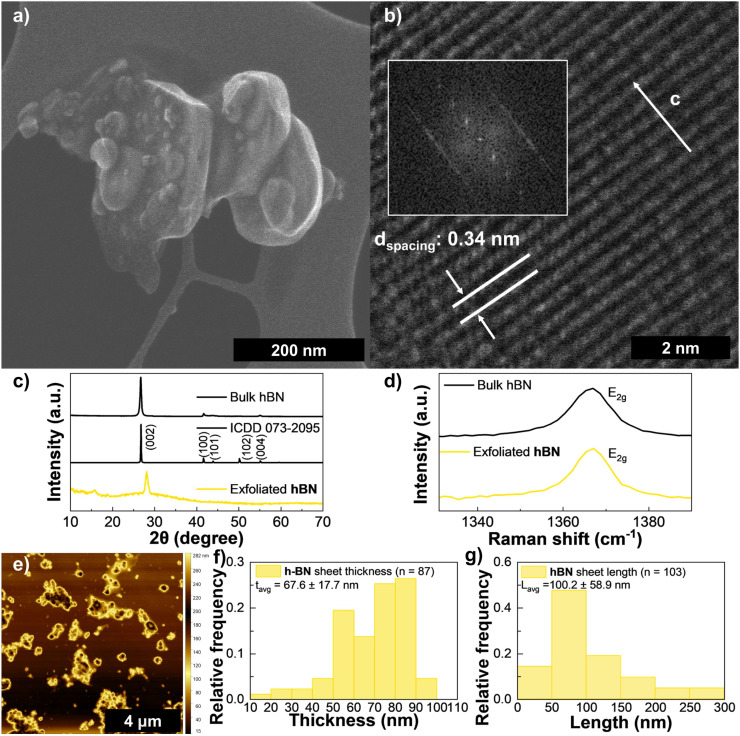
Characterization of hBN ink: (a) HAADF-STEM of exfoliated hBN, (b) HAADF-STEM of a stack along the *c* direction (the inset shows the power FFT patterns from the HAADF-STEM image), (c) XRD spectra for hBN (bottom), reference hexagonal boron nitride pattern ICDD 073-2095 (middle), and bulk hBN flakes (top), (d) Raman spectroscopy of exfoliated hBN (bottom) and bulk hBN flakes (top), (e) AFM scan of drop-casted ink diluted in IPA. (f) Distribution of hBN flake thickness *via* AFM, and (g) distribution of hBN flake length *via* TEM, the error in these reflects one standard deviation.

The HAADF-STEM imaging in Fig. 3(a) and (b) show LPE hBN flakes and a stack of hBN layers along the *c* axis with *d*-spacing of 0.34 nm similar to its theoretical value of 0.33 nm.^32^ A similar interlayer spacing of 0.32 nm was calculated from the XRD data in the bottom plot of Fig. 3(c) showing no signs of damage to the crystal structure of the precursor bulk powder. A decreasing intensity and broadening of the (002) plane feature is observed, compatible with the reduction of large bulk material into smaller less oriented crystallites. Raman spectra of LPE hBN and its precursor powder are shown in Fig. 3(d) bottom and top plots, respectively. Boron nitride only has a G band corresponding to an E_2g_ peak. This peak blue-shifts to higher wavenumbers in BN nanosheets with decreasing thickness, up to around 1370 cm^−1^ for monolayer hBN when bound to a substrate.^57,58^ The E_2g_ peak is centered at 1366.6 ± 0.3 cm^−1^ in the hBN sample, expected for exfoliated hBN, and at 1366.3 ± 0.3 cm^−1^ in the hBN powder, consistent with bulk hBN crystals.^57,58^ However this difference is at the maximum theoretical spectral resolution of the Raman microscope (0.3 cm^−1^) so an accurate comparison is not possible. From AFM imaging like Fig. 3(e) an average thickness of 68 nm (*n* = 87) (Fig. 3(f)) was obtained, the highest of all materials and from TEM and average 100 (*n* = 21) nm length was measured (Fig. 3(g)). This outcome is attributed to the high viscosity of Cyrene, which dampens the cavitation effects during sonication, leading to less effective delamination of the hBN layers.^9,25,43,52^

### 3.2 Transition metal oxides: V_2_O_5_ and MoO_3_

Inks of V_2_O_5_ consistently reached higher concentrations than the other materials and no meaningful sedimentation was observed over long term storage. A maximum yield of 2.67% (*C*_f_ = 1.87 mg mL^−1^) was achieved at a CF rate of 6000 rpm.

**Fig. 4:**
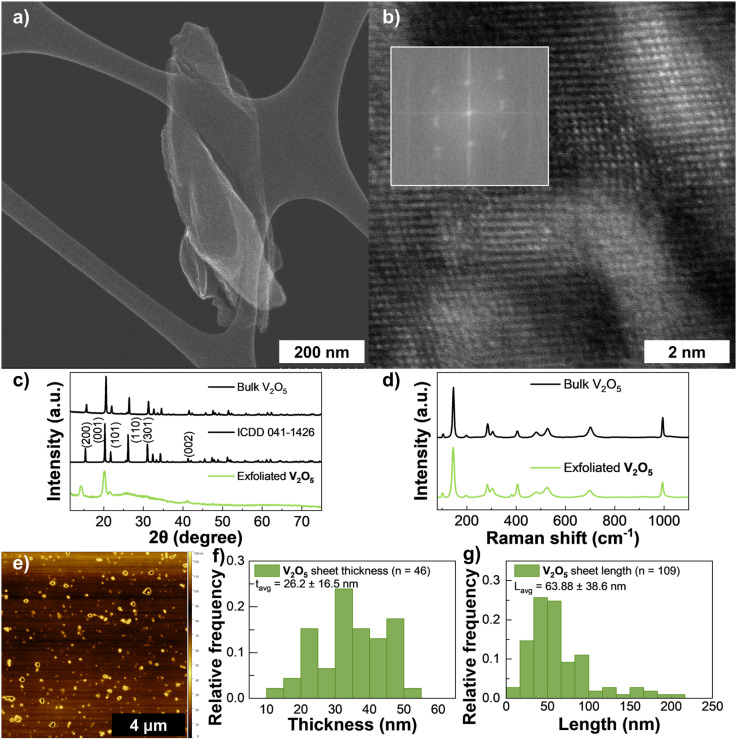
Characterization of V_2_O_5_ ink: (a) HAADF-STEM of a large, exfoliated V_2_O_5_ sheet, (b) HAADF-STEM of the top-view of a nanosheet (the inset shows the power FFT patterns from the HAADF-STEM image), (c) XRD spectra for V_2_O_5_ (bottom), reference vanadium pentoxide pattern ICDD 041-1426 (middle), and bulk V_2_O_5_ flakes (top), (d) Raman spectroscopy of exfoliated V_2_O_5_ (bottom) and bulk V_2_O_5_ flakes (top), (e) AFM scan of drop-casted ink diluted in IPA. (f) Distribution of V_2_O_5_ flake thickness *via* AFM, and (g) distribution of V_2_O_5_ flake length *via* TEM, the error in these reflects one standard deviation.

V_2_O_5_ inks were less prone to agglomeration as observable in the HRTEM images in Fig. 4(a) and (b). The XRD pattern in Fig. 4(c) shows spectra for the filtered sample, bulk powder and the reference pattern for bulk V_2_O_5_ (ICDD 041-1426). Bulk V_2_O_5_ usually contains several peaks and at lower centrifugation speeds (Fig. S9(c)) some of these features can be seen at 2*θ* = 15° (200), 20° (100), 21.5° (101), 26° (110), 31° (400), 32° (011), (310), 41° (002), 45° (411) and 47° (600).^59,60^ At higher CF rates most peaks are eliminated and the remaining (001) and (002) reflection planes become broader and less intense suggesting a preferential orientation along this direction.^60^ Less intense reflections are also still visible at 2*θ* = 15° and 27°, corresponding to the (200) and (101) planes, respectively, which might indicate a small presence of the bulk material. Given the broadening of main peaks and disappearance of most secondary planes, it is likely that a longer exfoliation time will improve the exfoliation quality. The crystallite size and *d*-spacing were calculated at 2.15 nm and 4.31 Å respectively which is consistent values reported in literature.^60^ Both the powder (top) and the sample (bottom) Raman spectra are shown on Fig. 4(d). The two spectra exhibit the characteristic peaks of α-V_2_O_5_, a orthorhombic polymorph of V_2_O_5_, with the top spectra showing peaks at 102 cm^−1^ (A_1g_), 145 cm^−1^ (B_1g_ + B_2g_ + B_3g_), 197 cm^−1^ (A_1g_ + B_2g_), 283 cm^−1^ (B_1g_ + B_3g_), 304 cm^−1^ (A_1g_), 406 cm^−1^ (A_1g_), 482 cm^−1^ (A_1g_), 525 cm^−1^ (A_1g_), 699 cm^−1^ (B_1g_ + B_3g_), 994 cm^−1^ (A_1g_ + B_2g_).^61–63^ Raman bands above 450 cm^−1^ are due to V–O bond-stretching.^61–63^ The Raman modes below 400 cm^−1^ are related to angle-bending vibrations. An extra small peak in the V_2_O_5_ sample (top spectra) can be seen at 381 cm^−1^, which can be attributed to either an E_g_ mode of V_2_O_3_, or an A_g_ + B_g_ mode of VO_2_ (M1).^61–63^ The bottom spectra of the V_2_O_5_ powder also shows the characteristic peaks of α-V_2_O_5_, and its peak centres are shifted 1–2 cm^−1^ towards lower wavenumbers. This shift to lower wavenumbers is expected for thin-films of α-V_2_O_5_ as their thickness decreases when compared to bulk α-V_2_O_5_ (Fig. S12).^64^ Additional samples prepared by spray-coating the ink onto glass required high temperatures to remove residual Cyrene (Fig. S8). Notably, the formation of V_4_O_9_ was observed when deposition was carried out on substrates heated to 300 °C. This is visible by the appearance of bands in the spectrum of Fig. S11 at 759 cm^−1^, 907 cm^−1^ (with a shoulder at 892 cm^−1^), and 949 cm^−1^.^62^ Still, for samples prepared at room temperature by filtering the inks, these bands are absent, confirming the presence of only α-V_2_O_5_. An absorption coefficient of 3.36 mg^−1^ mL cm^−1^ was calculated (Fig. S4(d)) and the exfoliated material had an average thickness of 26 nm (*n* = 46) determined *via* AFM statistics, and an average length of 64 nm (*n* = 109) determined from TEM statistics (Fig. 4(f) and (g), respectively).

Many layered metal oxides are commonly found in configurations of mixed valencies, requiring interlayer ions to balance their overall charge. As such their exfoliation is achieved by replacing these ions with larger species which induces the cleavage and separation of layers.^65^ However, some TMOs such as MoO_3_ are only found in single valence configuration and cannot be exfoliated this way. Still, as shown in previous studies their layers can still be peeled apart through LPE in solvents like NMP and other alcohols.^65,66^ LPE yields of MoO_3_ are generally lower than 1% with few reports going as high as 5.7% using low centrifugation speeds of 500 rpm over longer periods of time.^66^ We found that with Cyrene and a CF rate of 1500 rpm for 90 minutes it was possible to achieve a yield of 4.0% at an initial concentration of 50 mg mL^−1^ and 10 h of ultrasound bath (see Table S1). This yield quickly drops to 2.4, 1.7 and 0.68% at CF rates of 3000, 4500 and 6000 rpm, respectively. All characterization was performed at the highest CF rate with a maximum final concentration of 0.34 mg mL^−1^ which exhibited a slight blue coloration seen in Fig. S5(a)–(c) and some sedimentation was observed after 48 h.

**Fig. 5:**
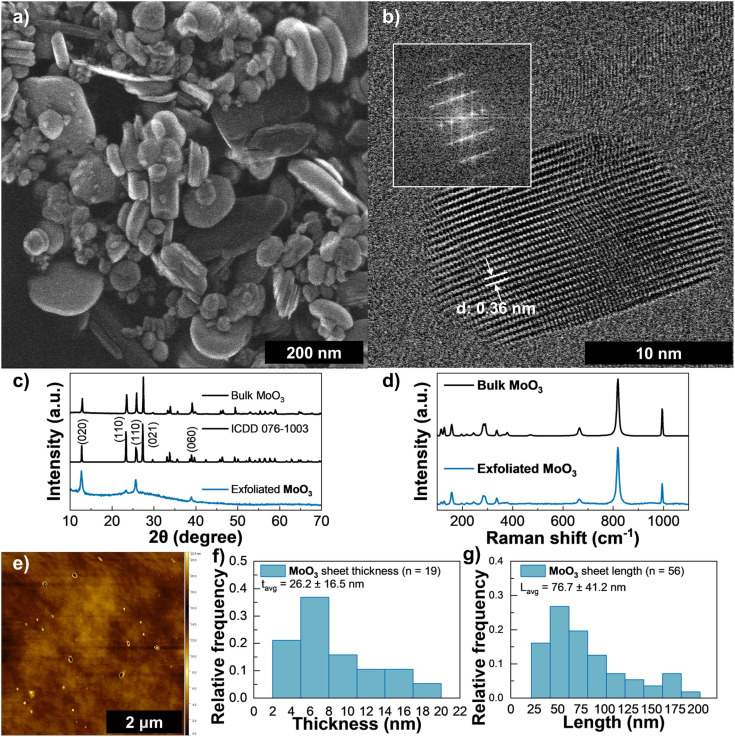
Characterization of MoO_3_ ink: (a) HAADF-STEM of a large, exfoliated MoO_3_ sheet, (b) HAADF-STEM of several layers stacked along the *c* direction (the inset shows the power FFT patterns from the HAADF-STEM image), (c) XRD spectra for MoO_3_ (bottom), reference molybdenum trioxide pattern ICDD 076-1003 (middle), and bulk MoO_3_ flakes (top), (d) Raman spectroscopy of exfoliated MoO_3_ (bottom) and bulk MoO_3_ flakes (top), (e) AFM scan of drop-casted ink diluted in IPA. (f) Distribution of MoO_3_ flake thickness *via* AFM, and (g) distribution of MoO_3_ flake length *via* TEM, the error in these reflects one standard deviation.

Multiple LPE MoO_3_ sheets and a stack consisting of less than 30 layers along the *c* direction are shown in Fig. 5(a) and (b), respectively, with a measured *d*-spacing of 0.36 nm which is in line with previous reports.^67,68^ The broadening of the bulk peaks in the XRD spectra (Fig. 5(c)) and disappearance of most reflections in Fig. S9(d) suggests some degree of exfoliation of the material with a preferential orientation along the (020) plane. Both Raman spectra in Fig. 5(d) show the typical characteristic peaks of crystalline α-MoO_3_. The bottom spectrum, measured on the filtered MoO_3_, is very similar to that obtained for exfoliated and vacuum filtered films of α-MoO_3_.^65^ Its characteristic peaks are found at 283 cm^−1^ (B_2g_, O=Mo=O wagging), 291 cm^−1^ (B_3g_, O=Mo=O wagging), 337 cm^−1^ (A_g_, O–Mo–O bending), 378 cm^−1^ (B_2g_, O–Mo–O scissoring), 664 cm^−1^ (B_3g_, O–Mo–O stretching), 819 cm^−1^ (A_g_, Mo=O terminal bond stretching), and 995 cm^−1^ (A_g_, Mo=O terminal bond stretching).^69,70^ Also present peaks at 117 cm^−1^ (B_2g_, translational rigid MoO_4_ chain mode), 127 cm^−1^ (B_1g_, translational rigid MoO_4_ chain mode), 158 cm^−1^ (B_2g_, translational rigid MoO_4_ chain mode), 197 cm^−1^ (B_3g_, O=Mo=O twist), 216 cm^−1^ (A_g_, rotational rigid MoO_4_ chain mode), and 245 cm^−1^ (B_1g_, O=Mo=O twist). The bottom spectrum from the MoO_3_ powder is very similar to the top spectra of the filtered MoO_3_, with a slight deviation in some peak centres and an extra shallow peak at 472 cm^−1^ (A_g_, O–Mo–O stretching and bending). Both spectra are consistent with polycrystalline or powdered α-MoO_3_ due to the relative intensity of the peaks at ∼995 cm^−1^ when compared to the ones at ∼664 cm^−1^ and ∼819 cm^−1^, and due to the splitting of the peak at ∼290 cm^−1^.^70^ The bands at Raman shifts below 400 cm^−1^ are consistent with a monolayered α-MoO_3_ sample, particularly the Raman peak at 115 cm^−1^ which arises from rigid chain Raman modes and does not appear in bulk α-MoO_3_.^70^ Dieterle *et al.*^71^ previously showed that the stoichiometries of MoO_3−*x*_, determined through the assessment of the material's oxygen vacancy-dependent band gap with diffuse reflection UV/vis spectroscopy, are directly proportional to the ratios of the Raman band intensities *I*_285_/*I*_295_ of the wagging modes at ∼285 cm^−1^ (B_2g_) and ∼295 cm^−1^ (B_3g_).^65,71^ The peak deconvolutions of the B_2g_ and B_3g_ bands are shown in Fig. S13 for both samples. In both spectra, there is a red shift of the B_3g_ peak arising from the presence of oxygen vacancies.^71^ For the filtered MoO_3_, the *I*_285_/*I*_295_ ratio was found to be 1.31, yielding a true stoichiometry of MoO_2.95_, while for the MoO_3_ powder this was 0.99, also resulting in a stoichiometry of MoO_2.95_.^71^ Given the direct proportionality of the *I*_285_/*I*_295_ ratio with the oxygen/metal atomic ratio, we can infer the powder has a negligibly lower oxygen fraction than the filtered sample, which can be attributed to the larger size of the nanosheets of the exfoliated material when compared with the powder.^65,71^ The absence of side peaks that would otherwise arise from oxygen vacancies around the 995 cm^−1^ Mo=O terminal stretching bonds, together with the stoichiometries determined above, demonstrate that the ink preparation method does not introduce more defects in the exfoliated material and may slightly reduce them.^65^ This contrasts with previously reported works of liquid exfoliation of MoO_3_ using 2-propanol (IPA) to produce nanosheets, where the solution processing reduced the stoichiometry of the MoO_3_ material from 2.96 in the raw powder to 2.94 in the nanosheets.^65^ AFM imaging (Fig. 5(e) and (f)) revealed an average thickness of 26 nm (*n* = 19), confirming the multi-layer nature of MoO_3_ and in TEM these showed an average flake length of 76.7 nm (*n* = 56) (Fig. 5(g)). As with the previous materials, Cyrene exfoliation yields content with higher thickness than reported for less viscous solvents such as 2-propanol (IPA).^65^

### 3.3 Transition metal dichalcogenides: MoS_2_ and WS_2_

**Fig. 6:**
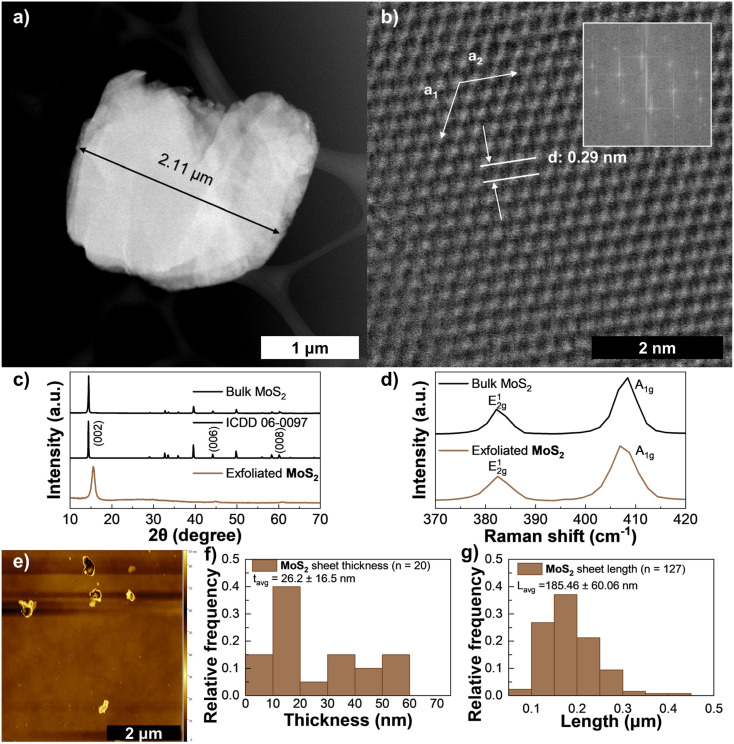
Characterization of MoS_2_ ink: (a) HAADF-STEM of a large, exfoliated MoS_2_ sheet, (b) HAADF-STEM of a top-view of a nanosheet (the inset shows the power FFT patterns from the HAADF-STEM image), (c) XRD spectra for MoS_2_ (bottom), reference MoS_2_ pattern ICDD 06-0097 (middle), and bulk MoS_2_ flakes (top), (d) Raman spectroscopy of exfoliated MoS_2_ (bottom) and bulk MoS_2_ flakes (top), (e) AFM scan of drop-casted ink diluted in IPA. (f) Distribution of MoS_2_ flake thickness *via* AFM, and (g) distribution of MoS_2_ flake length *via* SEM, the error in these reflects one standard deviation.

MoS_2_ was the material that consistently reached the highest concentrations, while also exhibiting long term stability and less precipitation than other materials. It was also stable when diluted in up to 1 : 20 v/v (Cyrene ink : IPA or ethanol) which facilitated its deposition at lower temperatures. It showed the highest repeatable yield of 3.8% (*C*_f_ = 1.9 mg mL^−1^) at a centrifugation rate of 6000 rpm increasing to 8.8% (*C*_f_ = 4.4 mg mL^−1^) at 1500 rpm. A large, exfoliated flake is shown in Fig. 6(a) and in panel (b) a top-view shows of the structure shows a well-coordinated hexagonal lattice without defects from the production process, typical of H2 MoS_2_.^13–15,72^ The measured *d*-spacing along the *c*-axis is coherent with other reports and shows no signs of significant damage caused to the structure during production.^73^ A periodic stacking along the *c* direction is visible in the XRD plot in Fig. 6(c), represented by the (002), (004), (006) and (008) reflections at 2*θ* = 15.4°, 31°, 44° and 61°, respectively. In Fig. S9(e) it is clearly shown the broadening of the main (002) peak for increased CF ratio, indicating smaller crystallite sizes as expected from the exfoliation process.^74–76^ As reported by,^21^ the spectra for MoS_2_ nanosheets exhibits a peak around 2*θ* = 14° and a broader feature between 20 and 30° which agrees with the results obtained for the LPE MoS_2_ in Cyrene. The produced MoS_2_ had a crystallite size of 1.8 nm (with a FWHM of 2*θ* = 0.91° measured at 2*θ* = 15.35°). The typical E^1^_2g_ and A_1g_ Raman peaks of MoS_2_ obtained with the 532 nm laser are presented in Fig. 6(d), which blue-shift and red-shift, respectively, when going from bulk samples to single-layer samples.^61,77,78^ Following the Lorentzian fitting of the peaks (see Fig. S14 and S15) the E^1^_2g_ and A_1g_ peaks of the filtered sample (bottom) are found at 382.6 cm^−1^ and 407.6 cm^−1^, respectively, while in the MoS_2_ powder (top) the E^1^_2g_ and A_1g_ peaks are found at 382.7 cm^−1^and 408.0 cm^−1^. This results in a Raman shift difference of 25.0 cm^−1^ for the exfoliated MoS_2_ sample, indicating the sample is at least 6 layers thick, and 25.3 cm^−1^ for the MoS_2_ powder, confirming the precursor powder as a bulk material.^77–80^ The excitonic properties (Fig. S6(d)) of MoS_2_ are visible in the features at *λ* = 591 nm and *λ* = 657 nm which are commonly associated with few-layer 2H–MoS_2_ and indicate no transition towards the metallic 1T phase.^76,81–84^ The average thickness of the exfoliated MoS_2_ sheets measured in AFM (Fig. 6(e) and (f)) was 26 nm (*n* = 20) and the length measured from SEM imaging (see Fig. S6(g)) averaged 186 nm (*n* = 127).

**Fig. 7:**
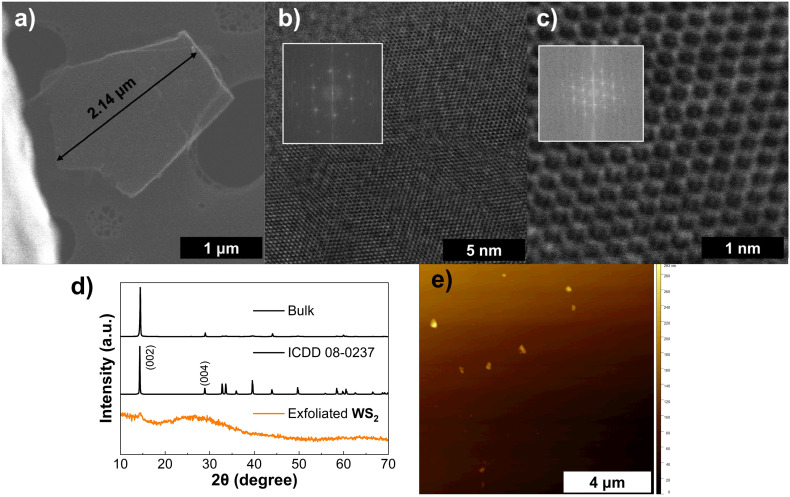
Characterization of WS_2_ ink: (a) HAADF-STEM of a large, exfoliated WS_2_ sheet, (b) and (c) HAADF-STEM of a top-view of a nanosheet (the inset shows the power FFT patterns from the HAADF-STEM image) at different magnifications, (d) XRD spectra for WS_2_ (bottom), reference WS_2_ pattern ICDD 08-0237 (middle), and bulk MoS_2_ flakes (top), (e) AFM scan of drop-casted ink diluted in IPA.

WS_2_, like MoS_2_, belongs to the family of layered TMDs, and both commonly adopt a 2H (hexagonal) crystal structure, space group *P*6_3_/*mmc* (no. 194). HRTEM imaging in Fig. 7(a) shows a large exfoliated sheet with 2H WS_2_ with its characteristic hexagonal structure shown in high magnification in (Fig. 7(b) and (c)). In both panels the structure show no damage from the LPE process. From the XRD pattern in Fig. 7(d) for this material a similar trend to previous materials is observed with a broadening of peaks as the CF rate was increased (Fig. S9(f)) as well as the disappearance of additional reflections from the bulk material. Like MoS_2_ a preference for the (001) reflections is seen in agreement with reports on exfoliated few-layer WS_2_.^85,86^ AFM imaging (Fig. 7(e)) showed flakes with an average height of 49.2 nm indicating predominantly multi-layered content and TEM imaging yielded an average flake length of 318 nm. A low extinction coefficient of 0.97 mL mg^−1^ cm^−1^ was obtained however due to ink instability and low exfoliation yields at high CF rates (shown in Fig. S7).

In a lab-scale setting the concentration of inks increased with exfoliation time, suggesting the potential for even higher yields and making Cyrene a strong candidate for integration into scalable production methods such as shear mixing.

## 4. Conclusions

The liquid-phase exfoliation of two-dimensional materials using Cyrene and low-power bath sonication has demonstrated the successful production of stable dispersions. This confirms Cyrene as an effective solvent for exfoliating 2DMs, including transition metal dichalcogenides (TMDs) and transition metal oxides (TMOs), yielding primarily few-to multi-layer structures.

Graphene inks produced multilayer flakes (typically >7 layers), confirmed through Raman spectroscopy and atomic force microscopy, with minimal oxidation observed by X-ray diffraction. These inks can be redispersed in other solvents, enhancing their versatility for various deposition techniques. Hexagonal boron nitride (hBN) inks reached concentrations up to 2.3 mg mL^−1^. Raman, XRD, and AFM analyses confirmed successful exfoliation into multilayer sheets, though dispersion stability remains a challenge. Vanadium pentoxide (V_2_O_5_) inks initially achieved high concentrations but showed long-term instability above 70 mg mL^−1^, with agglomeration and color changes. Characterization confirmed the presence of α-V_2_O_5_ and some V_4_O_9_ after high-temperature processing. MoS_2_ inks demonstrated excellent stability and processability, even when diluted in alcohols. Characterization confirmed few-layer flakes, and UV-vis spectroscopy revealed characteristic excitonic peaks, making MoS_2_ highly suitable for electronics and energy applications. MoO_3_ inks were successfully exfoliated, with few-layer structures confirmed *via* Raman and XRD. Moderate dispersion stability was observed, with some sedimentation after 48 h. AFM and SEM analysis showed flake dimensions consistent with literature, supporting its potential in sensors and catalysis.

With further optimization, including longer sonication and solvent-switching methods, Cyrene offers a sustainable and effective route for 2DM ink production across various applications.

## Author contributions

Pedro Moreira – methodology, investigation, formal analysis, conceptualization and writing – original draft; João Mendes, Tomás Calmeiro, Daniela Nunes, David Carvalho and Adam Kelly – investigation and formal analysis; Hugo Águas, Elvira Fortunato and Rodrigo Martins – funding acquisition and writing – review editing; Joana Vaz Pinto, João Coelho and Emanuel Carlos – funding acquisition, supervision, visualization, validation and writing – review & editing. All authors have read and agreed to the published version of the manuscript.

## Conflicts of interest

The authors declare no competing interests.

## Supplementary Material

NA-OLF-D5NA00576K-s001

## Data Availability

The data supporting this article has been included as part of the supporting information (SI). Supplementary information is available. See DOI: https://doi.org/10.1039/d5na00576k.
