# Supplementary material for: Green exfoliation of 2D nanomaterials using cyrene as a solvent
Source: Nanoscale Adv. 2025 Oct 14;7(23):7754–67. doi: 10.1039/d5na00576k (PMC12536451; doi:10.1039/d5na00576k)
Supplement: NA-007-D5NA00576K-s001 [file NA-007-D5NA00576K-s001.pdf]

Supplementary Information (SI) for Nanoscale Advances.
This journal is © The Royal Society of Chemistry 2025

Supporting Information

# Green exfoliation of 2D nanomaterials using Cyrene as a solvent

Pedro Moreira[1], João Mendes[1], Tomás Calmeiro[1], Daniela Nunes[1], David Carvalho[1], Adam Kelly[1], Hugo Águas[1], Elvira Fortunato[1], Rodrigo Martins[1], Joana Vaz Pinto[1], João Coelho[2*], Emanuel Carlos[1*]

[1]CENIMAT|i3N, Department of Materials Science, School of Science and Technology, NOVA University Lisbon and CEMOP/UNINOVA, Campus de Caparica, 2829-516 Caparica, Portugal

[2]Department of Condensed Matter Physics, Institute of Materials Science of Seville, University of Seville—CSIC, Avenida Reina Mercedes SN, 41012 Seville, Spain

*e-mail: e.carlos@fct.unl.pt; jmesquita@us.es

# 1. Additional concentration optimization results

In Figure S1 the values of final concentration ($C_f$) as a function of initial concentration ($C_i$), ultrasound (US) exfoliation time and centrifugation (CF) rates are shown (panels (a), (b), and (c), respectively). In the first study a stagnation of final concentration was observed, typically above $C_i$ of 70 mg mL$^{-1}$ with some materials $C_f$ even decreasing afterwards. In the second study $C_f$ increased with US time but a greater increase occurs in the earlier US hours and therefore a optimal exfolation time was chosen for each material beyond which even if $C_f$ keeps increasing it becomes less meaningful. In the third study (panel (c)) considerable amounts of material were lost by increasing the CF rates and yield rates were calculated from the weighed concentration after each centrifugation step. The optimized synthesis settings for each 2D material (2DM) is shown in Table S1 as well as the yield observed at each CF rate. Here, yield refers to the ratio of the ink concentration obtained at the centrifugation speeds listed in Table S1 to the initial precursor concentration used.

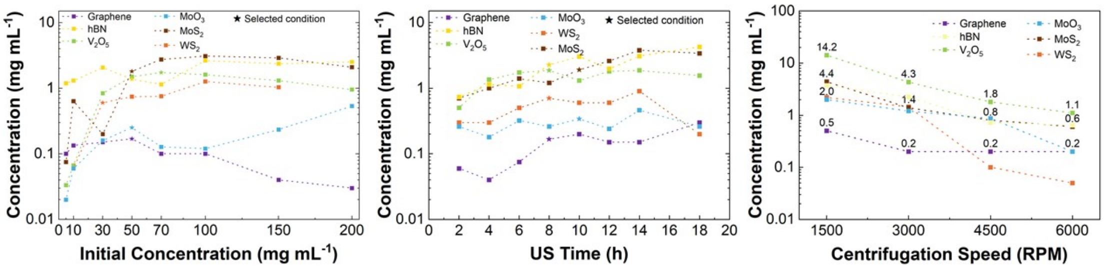


Figure S1 – Concentration achieved for each material (graphene, hBN, $V_2O_5$, $MoO_3$, $WS_2$, $MoS_2$) of (a) initial precursor powder concentration (5-200 mg mL$^{-1}$), (b) ultrasound exfoliation time (2-18 hours), and (c) centrifugation rate (1500, 3000, 4500 and 6000 rpm). Measurement error is below symbol size due to high-resolution mass balance and second precision timing.

Table S1: Optimized concentration obtained for each material and procedure settings used. All samples were also centrifuged for 30 minutes at 1000 rpm prior to the final CF step.

| | Initial concentration | US time | Final concentration CF rate = 1500 rpm | | Final concentration CF rate = 6000 rpm | |
|---|---|---|---|---|---|---|
| Material | (mg mL$^{-1}$) | (h) | (mg mL$^{-1}$) | Yield (%) | (mg mL$^{-1}$) | Yield (%) |
| Graphene | 50 | 10 | 0.5 | 1.00 | 0.2 | 0.40 |
| hBN | 50 | 8 | 3.725 | 7.45 | 2.27 | 4.54 |
| $V_2O_5$ | 70 | 8 | 14.2 | 20.29 | 1.87 | 2.67 |
| $MoO_3$ | 50 | 10 | 2 | 4.00 | 0.34 | 0.68 |
| $MoS_2$ | 50 | 10 | 4.4 | 8.80 | 1.9 | 3.80 |
| $WS_2$ | 30 | 8 | 2.2 | 7.33 | 0.9 | 3.00 |

The following Table S2 shows relevant publications on the LPE of various 2D materials in a wide range of solvents and exfoliation methods including ultrasound bath, probe sonication (PS) and ion intercalation (II) assisted exfoliation.

Table S2 – Overview of 2DMs by liquid phase exfoliation methods.

| Material / Method | Ink Stability (days)** | Solvent | Concentration (mg mL$^{-1}$) | Layers | Length (nm) | Application | Ref. |
|---|---|---|---|---|---|---|---|
| Graphene / US | 21 | NMP | 0.01 | mono to few-layer | < 100 | - | 2008[1] |
| $MoS_2$ / PS | > 4 | NMP | 0.3 | few-layer | 170 | - | 2011[2] |
| $WS_2$ / PS | > 4 | NMP | 0.15 | few-layer | 250 | - | 2011[2] |
| hBN / PS | > 4 | IPA | 0.06 | few-layer | 960 | - | 2011[2] |
| $MoS_2$ / ME | - | N.A. | - | monolayer | > 1000 | TFT semiconductor | 2011[3] |
| hBN / PS | - | Water + PVA | - | few-layer | 1300 | Polymer composite | 2013[4] |
| Graphene / PS | > 30 | DMF | 0.1 | mono to few-layer | - | - | 2014[5] |
| $MoO_3$ / PS | 12 | IPA | 0.17 | few-layer | > 1100 | Supercapacitor electrodes | 2014[6] |
| $MoS_2$ / II | 3 | Water* | - | monolayer | 10000 | Inkjet printing | 2014[7] |
| $WS_2$ / II | 3 | Water* | - | monolayer | 10000 | Inkjet printing | 2014[7] |

| | | | | | | | |
|---|---|---|---|---|---|---|---|
| hBN / US | - | DMF / NMP / DMSO | - | bilayer | < 1000 | - | 2014[8] |
| $MoS_2$ / US | - | DMF / NMP / DMSO | - | bilayer | < 1000 | - | 2014[8] |
| $MoS_2$ / PS | > 14 | Chloroform + acetonitrile | - | Few layer | 200 | Humidity sensor | 2014[9] |
| $WS_2$ / US | - | DMF / NMP / DMSO | - | bilayer | 80-100 | - | 2014[8] |
| Graphene / PS | - | NMP | <1.8 | few layer | 920 | - | 2015[10] |
| Graphene / II | - | Water* | - | few layer | < 1000 | - | 2015[11] |
| $MoS_2$ / II | - | BuLi + hexane | - | monolayer | 300 | - | 2015[12] |
| Graphene / US | > 30 | NMP | <1 | few layer | < 5000 | - | 2015[13] |
| hBN / US | > 30 | NMP / IPA | <1 | few layer | < 1500 | - | 2015[13] |
| $MoS_2$ / US | > 30 | NMP / IPA / $H_2O$ + SDS | <1 | few layer | < 1000 | - | 2015[13] |
| $WS_2$ / US | > 30 | $H_2O$ + SDS | <1 | few layer | < 800 | - | 2015[13] |
| Graphene / PS | > 150 | Water* | 0.5 | few layer | > 1000 | - | 2016[14] |
| $MoS_2$ / PS | - | NMP + DMF | 18 | multilayer | > 121 | Phototransistor | 2016[15] |
| $WS_2$ / PS÷ | - | Acetone + IPA | - | few-layer | 1200 | Humidity sensor | 2016[16] |
| Graphene / PS | - | Cyrene | 0.7 | few-layer | 1323 | - | 2017[17] |
| $V_2O_5$ / PS | > 7 | water + ethanol | 0.19 | - | 805 | Battery cathode | 2017[18] |
| Graphene / II | - | NMP* | 0.21 | multilayer | 420 | - | 2017[19] |
| $MoS_2$ / II | - | DMSO | 10 | bi to few-layer | 800 | - | 2018[20] |
| Graphene / PS | - | NMP | 0.32 | few-layer | 183 | - | 2018[21] |
| $MoS_2$ / II | - | N.A. | - | monolayer | 10000 | - | 2019[22] |

| | | | | | | | |
|---|---|---|---|---|---|---|---|
| Graphene / PS | - | $H_2O^*$ | - | bi to few-layer | < 1000 | - | 2019[23] |
| hBN / PS | - | $H_2O^*$ | - | few-layer | - | - | 2019[23] |
| $MoS_2$ / PS | - | $H_2O^*$ | - | bi to few-layer | - | - | 2019[23] |
| $WS_2$ / PS | - | $H_2O^*$ | - | few-layer | < 300 | - | 2019[23] |
| $MoS_2$ / PS | - | IPA + water | 1 | multilayer | 100-200 | Photodetector | 2020[24] |
| $WS_2$ / PS | - | IPA + water | 1 | multilayer | 100-200 | Photodetector | 2020[24] |
| $WS_2$ / $II^{\dagger}$ | - | EG* | N.A. | multilayer | > 10 | Photodetector | 2020[25] |
| Graphene / PS | - | NMP / DMF* | - | few- to multilayer | - | - | 2020[26] |
| Graphene / PS | > 120 | IPA / Ethanol / NMP | - | single- to multilayer | - | - | 2021[27] |
| $V_2O_5$ / II | - | NMP | N.A. | multilayer | > 100 | Battery cathode | 2021[28] |
| Graphene / PS | - | Cyrene | 1.22 | multilayer | - | Touch-screens | 2021[29] |
| Graphene / PS | - | NMP | 0.29 | multilayer | - | Touch-screens | 2021[29] |
| Graphene / PS | - | DMF | 0.10 | - | - | Touch-screens | 2021[29] |
| $WS_2$ / PS | - | Water* | N.A. | few-layer | < 500 | Resistor | 2023[30] |
| $V_2O_5$ / II | - | Water | 9.71 | multilayer | < 2000 | Battery cathode | 2023[31] |
| $MoS_2$ / US | - | Cyrene | N.A. | mono to few-layer | < 38 | - | 2023[32] |
| $WS_2$ / US | - | Cyrene | N.A. | mono to few-layer | < 21 | - | 2023[32] |
| $MoS_2$ / US | > 15 | NMP | 0.2 | few-layer | < 1000 | - | 2024[33] |
| hBN / US | > 15 | IPA / $H_2O$ | 0.2 | few-layer | < 500 | - | 2024[33] |
| $WS_2$ / US | > 15 | NMP | 0.5 | few-layer | < 500 | - | 2024[33] |
| $WS_2$ / US | > 15 | NMP | 0.5 | few-layer | < 500 | - | 2024[33] |

| | | | | | | | |
|---|---|---|---|---|---|---|---|
| Graphene / US | 7 | Cyrene | 0.2 | few to multi-layer | 445 | - | This work |
| hBN / US | 2 | Cyrene | 2.27 | multi-layer | 100 | - | This work |
| $V_2O_5$ / US | 30 | Cyrene | 1.87 | few-layer | 64 | - | This work |
| $MoO_3$ / US | 2 | Cyrene | 0.34 | few-layer | 77 | - | This work |
| $MoS_2$ / US | 30 | Cyrene | 1.9 | few-layer | 185 | - | This work |
| $WS_2$ / US | 2 | Cyrene | 0.9 | - | - | - | This work |

**Time that the ink remained stable without agglomeration and sedimentation. *Additives or surfactants were also used. [+]Processing/sonication time of 10 h or more was used

Figure S2 presents a systematic study of the effects of key processing parameters on the exfoliation and dispersion of graphene. Panel (a) illustrates the influence of increasing initial precursor concentration on the resulting dispersion. Panel (b) examines the effect of ultrasound bath time, while panel (c) shows variations in centrifugation rates. Panel (d) depicts the extinction-concentration relationship of the dispersed material, confirming a linear correlation. Panel (e) provides a schematic representation of the graphene structure, highlighting interlayer spacing. Panel (f) displays an SEM image of the exfoliated material, revealing its morphological characteristics. Finally in panel (g) a study of the viscosity of inks centrifuged at increasing rates and temperatures is provided along with the respective reverso Ohnsorges number (Z), a key parameter in assessing ink compatibility with printing techniques, described in detail elsewhere [34].

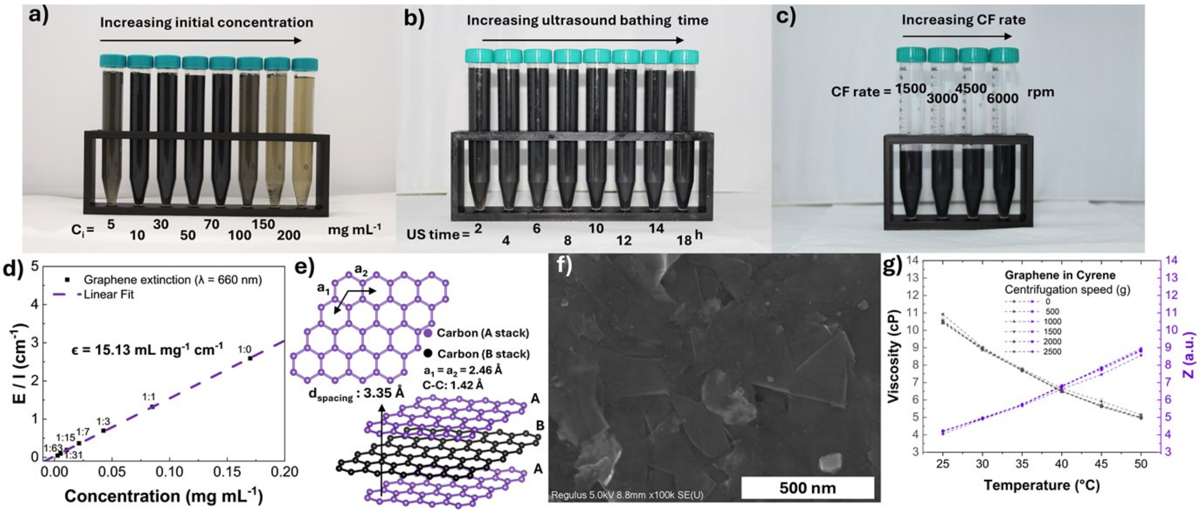


Figure S2 – (a) Effect of increasing initial precursor concentration on dispersion stability. (b) Influence of ultrasound bath duration on exfoliation. (c) Impact of different centrifugation rates on dispersion quality. (d) Extinction spectra and corresponding linear fit used to determine the extinction coefficient of graphene dispersions. (e) Atomic structure of graphene, highlighting interlayer spacing. (f) SEM image of exfoliated graphene flakes at a magnification of 100k. (g) Viscosity and reverse Ohnsorge number for graphene inks centrifuged at various rates and temperatures.

Figure S3 presents the investigation of the exfoliation of hexagonal boron nitride (hBN) under varying processing conditions. Panel (a) illustrates the effect of increasing initial precursor concentration on the LPE efficiency. Panel (b) examines the influence of ultrasound bath duration, while panel (c) shows the impact of different centrifugation rates. The absorbance-concentration spectrum in panel (d) is used to determine the extinction coefficient of hBN dispersions. Panel (e) provides a schematic representation of the hBN crystal structure, highlighting B–N bond lengths and interlayer spacing. Finally, panel (f) displays an SEM image of the exfoliated hBN material, revealing its morphological characteristics.

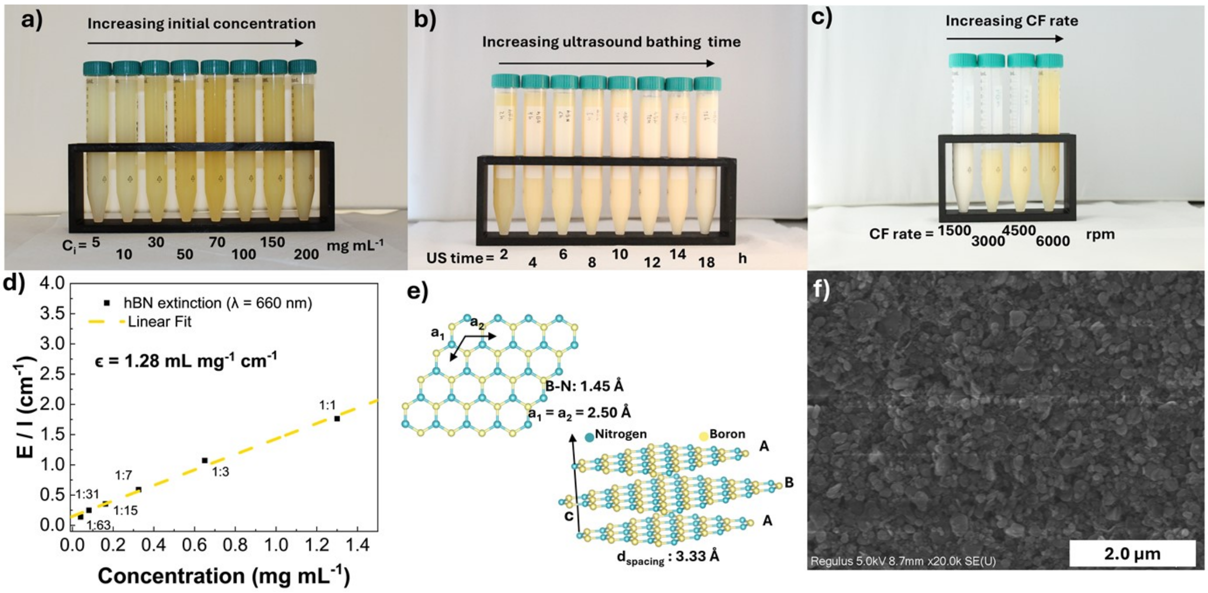


Figure S3 – Effect of increasing initial precursor concentration on dispersion stability. (b) Influence of ultrasound bath duration on exfoliation. (c) Impact of different centrifugation (CF) rates on dispersion quality. (d) Extinction spectra and corresponding linear fit used to determine the extinction coefficient of hBN dispersions. (e) Atomic structure of hBN, highlighting B–N bond lengths and interlayer spacing. (f) SEM image of exfoliated hBN flakes at a magnification of 20k.

Figure S4 presents the systematic study of exfoliated vanadium pentoxide ($V_2O_5$) dispersions under varying processing conditions. Panel (a) illustrates the effect of increasing initial precursor concentration on dispersion concentration. Panel (b) explores the impact of ultrasound bath duration, while panel (c) investigates the influence of different centrifugation (CF) rates. The absorbance spectra in panel (d) demonstrate the optical response of $V_2O_5$ dispersions used to determine the extinction coefficient. Panel (e) depicts the atomic structure of $V_2O_5$, highlighting bond lengths and interlayer spacing. Finally, panel (f) presents an SEM image of the exfoliated $V_2O_5$ material, revealing its morphology.

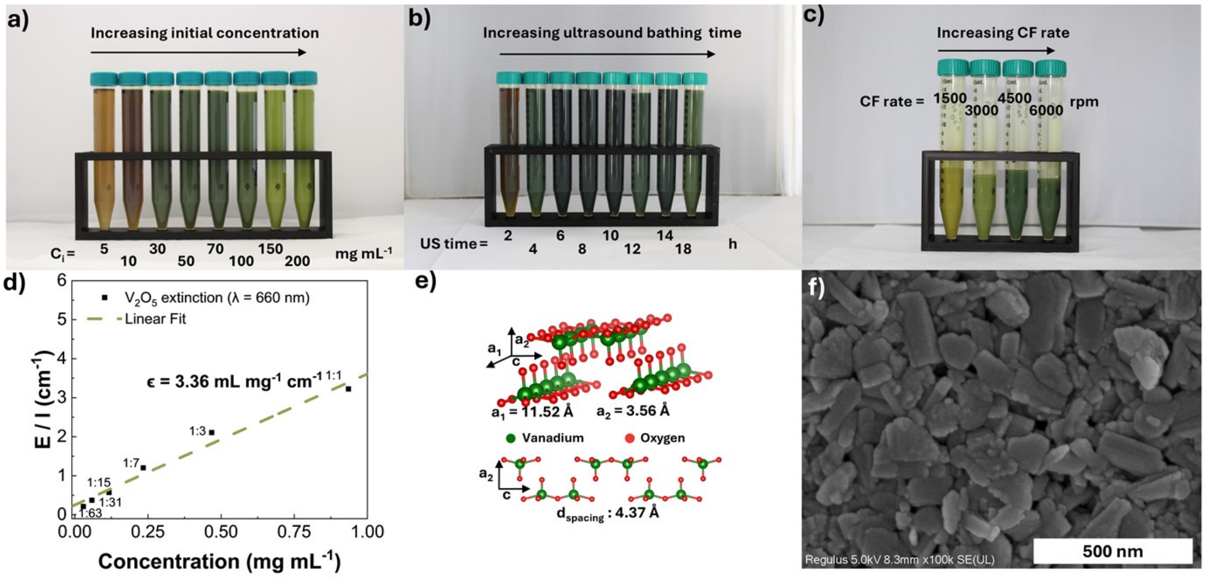


Figure S4 - Effect of increasing initial precursor concentration on dispersion stability. (b) Influence of ultrasound bath duration on exfoliation. (c) Impact of different centrifugation (CF) rates on dispersion quality. (d) Extinction spectra and corresponding linear fit used to determine the extinction coefficient of $V_2O_5$ dispersions. (e) Atomic structure of $V_2O_5$, highlighting bond lengths and interlayer spacing. (f) SEM image of exfoliated $V_2O_5$ flakes at a magnification of 100k.

Figure S5 presents the systematic study of exfoliated molybdenum trioxide ($MoO_3$) dispersions under different processing conditions. Panel (a) illustrates the effect of increasing initial precursor concentration on dispersion stability. Panel (b) examines the influence of ultrasound bath duration, while panel (c) explores the impact of different centrifugation (CF) rates. The absorbance spectra in panel (d) highlight the optical response of $MoO_3$ dispersions used to determine the extinction coefficient. Panel (e) provides a structural representation of $MoO_3$, detailing bond lengths and interlayer spacing. Finally, panel (f) presents an SEM image of the exfoliated $MoO_3$ material, revealing its morphology.

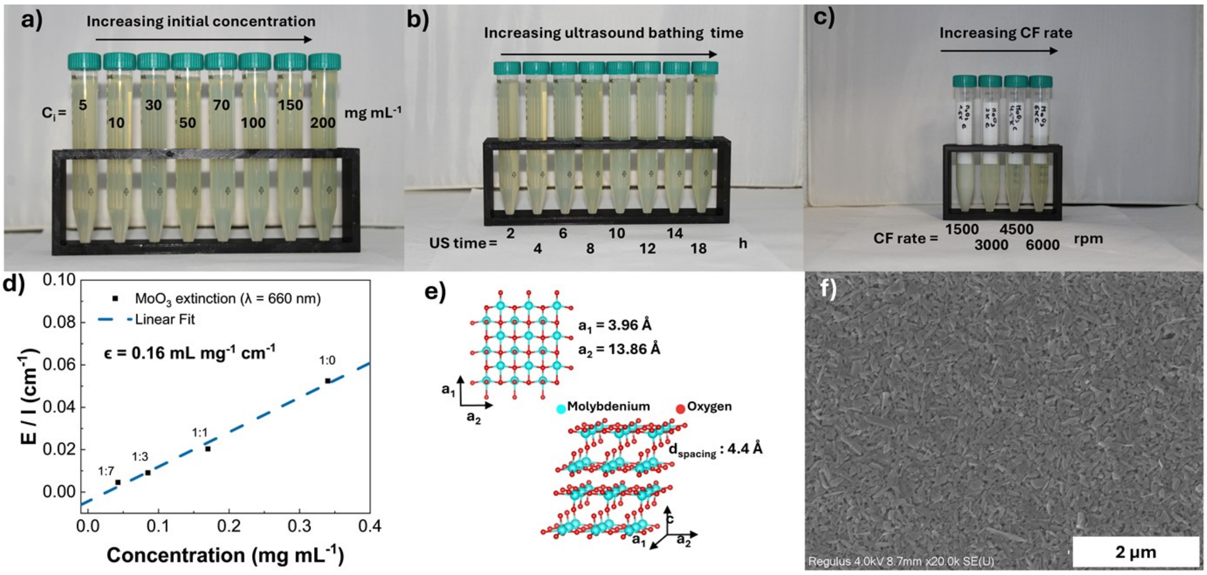


Figure S5 - Effect of increasing initial precursor concentration on dispersion stability. (b) Influence of ultrasound bath duration on exfoliation. (c) Impact of different centrifugation (CF) rates on dispersion quality. (d) Extinction spectra and corresponding linear fit used to determine the extinction coefficient of $MoO_3$ dispersions. (e) Atomic structure of $MoO_3$, showing bond lengths and interlayer spacing. (f) SEM image of exfoliated $MoO_3$ flakes at a magnification of 20k.

Figure S6 presents the study of exfoliated molybdenum disulfide ($MoS_2$) dispersions in cyrene under different processing conditions. Panel (a) shows the effect of increasing initial precursor concentration. Panel (b) investigates the influence of ultrasound bath time, while panel (c) examines the effect of different centrifugation rates on final ink concentration. The extinction spectra in panel (d) highlight the characteristic optical response of $MoS_2$, with the inset showing the linear correlation between concentration and absorbance, used to determine the extinction coefficient. Panel (e) illustrates the atomic structure of $MoS_2$, indicating Mo–S bond lengths and interlayer spacing. Finally, panel (f) presents an SEM image of the exfoliated $MoS_2$ flakes, showcasing their morphology and lateral dimensions.

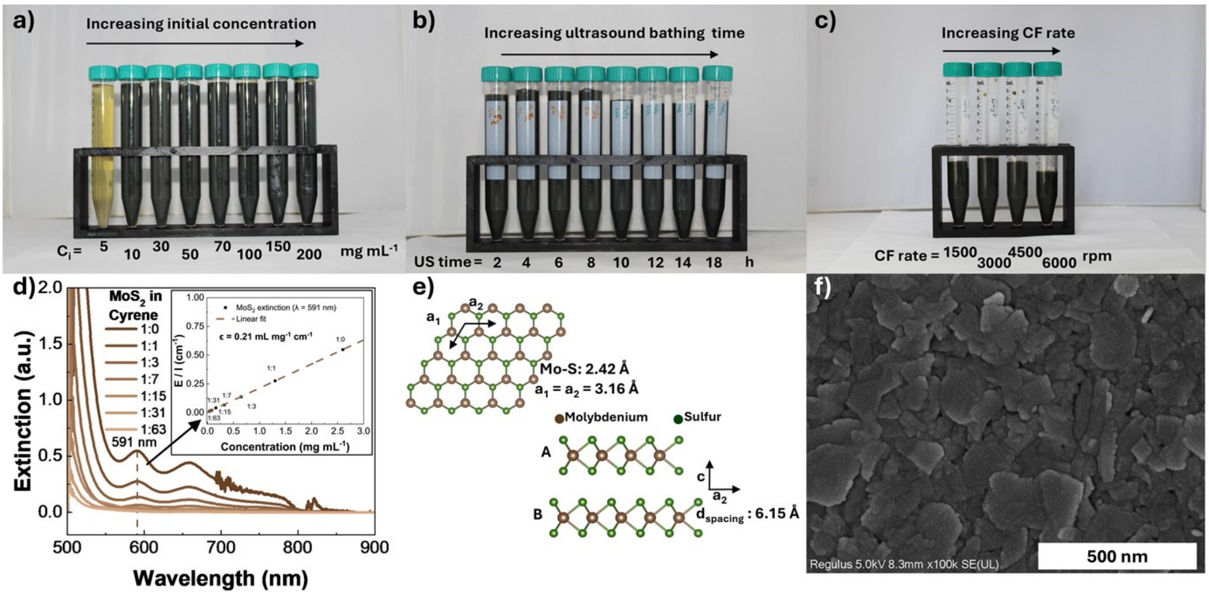


Figure S6 – Effect of increasing initial precursor concentration on dispersion stability. (b) Influence of ultrasound bath duration on exfoliation. (c) Impact of different centrifugation (CF) rates on dispersion quality. (d) Extinction spectra and corresponding linear fit used to determine the extinction coefficient of $MoS_2$ dispersions. (e) Atomic structure of $MoS_2$, showing Mo–S bond lengths and interlayer spacing. (f) SEM image of exfoliated $MoS_2$ flakes at a magnification of 100k.

In Figure S7 additional results pertaining to the LPE of $WS_2$ are shown. In panel (a) the inks produced during the optimization of initial concentration and in (b) inks from the optimization of exfoliation time. In panel (c) the 2H structure of a $WS_2$ is shown with characteristic bonding lengths. The extinction coefficient is calculated in Figure S7(d). HAADF-STEM imaging is shown in panels (e) and (f) in low and high magnifications, respectively, showing the typical flake size as well as the absence of defects in the processed sheets. Lastly in Figure S7(g) a filtration of an unstable ink is shown.

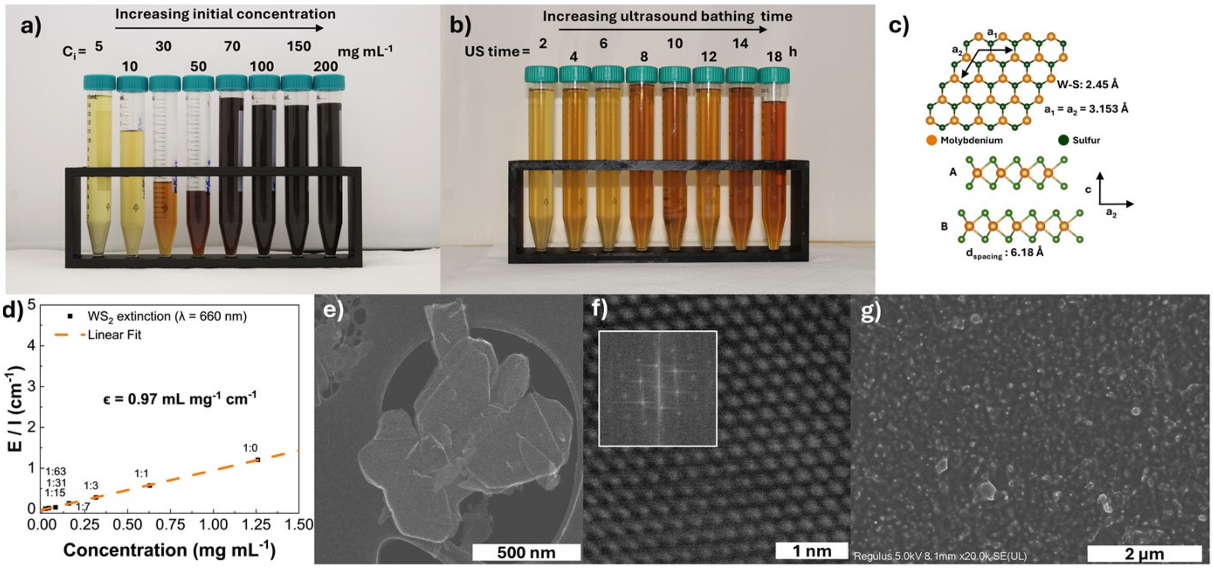


Figure S7 – (a) Effect of increasing initial precursor concentration on dispersion stability. (b) Influence of ultrasound bath duration on exfoliation. (c) Atomic structure of $WS_2$, showing W–S bond lengths and interlayer spacing. (d) Extinction spectra and corresponding linear fit used to determine the extinction coefficient of $WS_2$ dispersions. HAADF-STEM imaging of (e) exfoliated $WS_2$ sheets and (f) top-view of the hexagonal lattice, the inset shows the FFT. (g) SEM imaging of a filtered sample.

## 2. Cyrene DSC-TG

DSC-analysis of Cyrene was performed to assess the minimum temperature requirement for its removal from samples, shown in Figure S8. Analysis was conducted under Nitrogen:Air (80:20) atmosphere from 20 to 500 °C at a constant rate of 5 °C/min and shows a near complete loss of mass at 150 °C which is well below its reported boiling temperature of 220 °C.

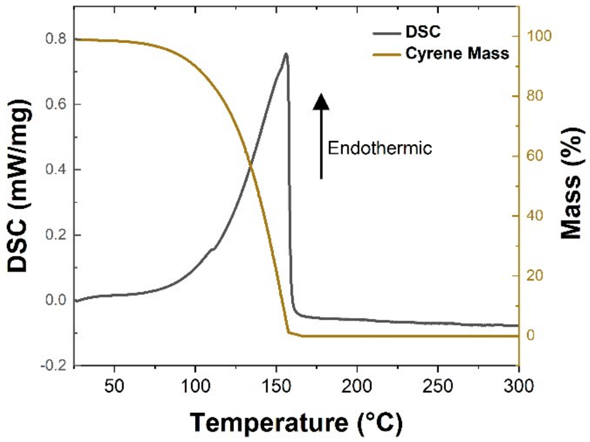


Figure S8 - DSC-TG analysis of cyrene showing a decrease of nearly all of its mass at around 150 °C, below its boiling point of 223 °C.

## 3. Additional XRD Results

XRD spectra was collected on a range of 2θ = 10 to 90° over a 15 minute runtime measurement using a Cu-κ anode and a λ of 1.54 Å. For all materials a decrease in intensity is observed as the CF rate is raised, associated with the lower amount of material deposited in the filter. Additionally, for all materials a broadening of main peaks is also observed which suggests an increase in exfoliated content. In lower concentration measurements a bump around 2θ = 26° is seen due to the background spectrum of the filter itself, shown in figure S9b).

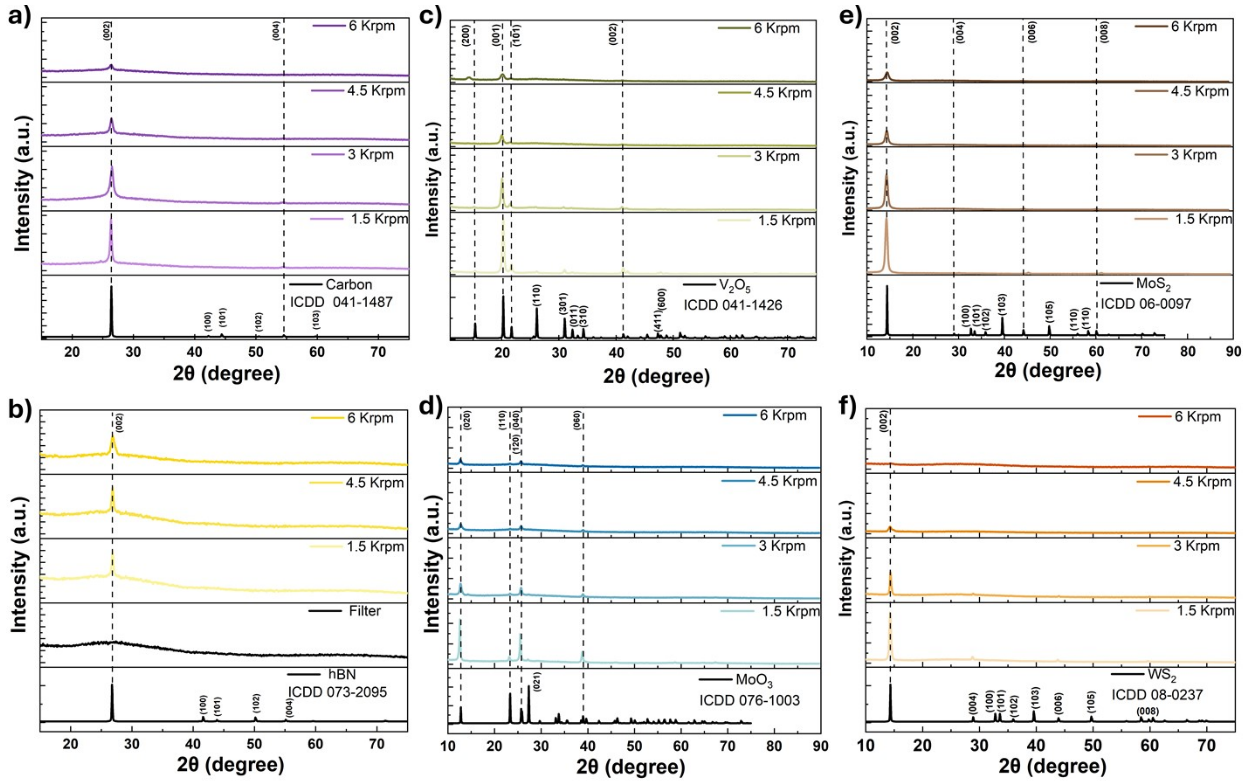


Figure S9 – XRD results for filtered samples at various CF rates: (a) graphene, (b) hBN, (c) $V_2O_5$, (d) $MoO_3$, (e) $MoS_2$ and (e) $WS_2$.

For every material the crystallite size decreased as CF was raised as seen in Table S3 suggesting a increased degrees of exfoliation. Additionally, the calculated d-spacing remained similar for all CF rates indicating no significant damage was induced on the structure of the materials.

Table S3 - Crystallite size calculated from Scherrer's equation for all materials at varying CF rates.

| | Crystallite Size | | | | d-spacing | | | |
|---|---|---|---|---|---|---|---|---|
| **Material** | 1500 RPM | 3000 RPM | 4500 RPM | 6000 RPM | 1500 RPM | 3000 RPM | 4500 RPM | 6000 RPM |
| **Graphene** | 5.14 | 2.86 | 3.30 | 2.71 | 3.30 | 3.24 | 3.25 | 3.29 |
| **hBN** | 5.10 | 4.87 | 4.86 | 2.25 | 3.20 | N.A. | 3.17 | 3.32 |
| **$V_2O_5$** | 2.80 | 2.41 | 2.14 | 1.75 | 4.29 | 4.33 | 4.34 | 4.31 |
| **$MoO_3$** | 4.19 | 5.15 | 2.49 | 1.25 | 6.90 | 6.80 | 6.79 | 6.82 |
| **$MoS_2$** | 4.29 | 4.13 | 3.28 | 2.15 | 5.80 | 6.00 | 5.81 | 5.98 |
| **$WS_2$** | 6.35 | 3.38 | 2.85 | 3.33 | 3.32 | 5.80 | 6.09 | 5.70 |

# 4. Additional Raman Results

Micro-Raman spectroscopy (Reinshaw inVia Raman microscope) was done to evaluate the quality of exfoliation of each 2DM in a Renishaw inVia Qontor confocal Raman Microscope by focusing either a 532 nm frequency doubled Nd:YAG DPSS excitation laser (Renishaw RL532C50) or a 633 nm HeNe Laser (Renishaw RL633) at varying power values, on the samples using a Leica Nplan 50× objective (NA 0.75, WD 0.37 mm) or a Leica Nplan 100× objective (NA 0.85) to achieve laser spots with sizes between 0.8 and 1.0 $\mu m^2$. An 1800 l/mm grating was used in all cases. The full spectra acquisition conditions for all samples are shown on Table S4.

Table S4 - Raman spectra acquisition conditions for all samples.

| Material | Laser wavelength (nm) | Grating (l/mm) | Lens | Laser power (mW) | Number of accumulations | Exposure time (s) |
|---|---|---|---|---|---|---|
| Graphene | 532 | 1800 | 100× | 3 | 10 | 10 |
| $V_2O_5$ | 532 | 1800 | 50× | 3 | 10 | 2 |
| $MoS_2$ | 532<br>633 | 1800 | 50× | 1.5<br>0.1 | 10 | 5 |
| $MoO_3$ | 532 | 1800 | 50× | 3 | 10 | 3 |
| hBN | 532 | 1800 | 100× | 3 | 10 | 2 |

The deconvolutions of the 2D peaks of the graphene flakes and graphene samples following four Lorentzian peak fits ($2D_{1B}$, $2D_{1A}$, $2D_{2A}$, and $2D_{2B}$) are shown on Figure S10. The graphene sample has a different shape to its 2D peak than the graphene flakes, with the latter peak shape being characteristic of graphite [35].

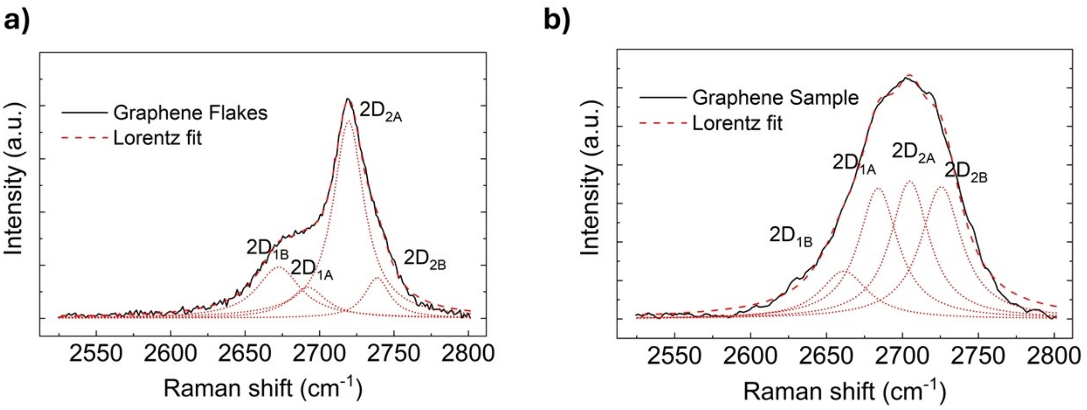


Figure S10 - Raman spectra of the precursor graphene flakes (left) and the graphene sample (right), both deconvoluted according to Lorentz peak fits.

The following Table S5 shows the positions and relative intensities of the characteristic graphene peaks suggesting few to multi-layered flakes with more than 5 layers [36-38]. The 2D band centre was taken as the centre of the $2D_{2A}$ peaks in both spectra.

Table S5 – Raman peak characteristics of the graphene flakes and graphene sample.

| Graphene | D band (cm$^{-1}$) | G band (cm$^{-1}$) | 2D band (cm$^{-1}$) | $I_D/I_G$ | $I_{2D}/I_G$ |
|---|---|---|---|---|---|
| Flakes | - | 1582 | 2720 | - | 0.66 |
| Sample | 1347 | 1582 | 2705 | 0.59 | 0.52 |

The $E_{2g}$ peak centers of the hBN powder and sample were obtained by fitting the peaks with Lorentz functions, as seen on Figure S10.

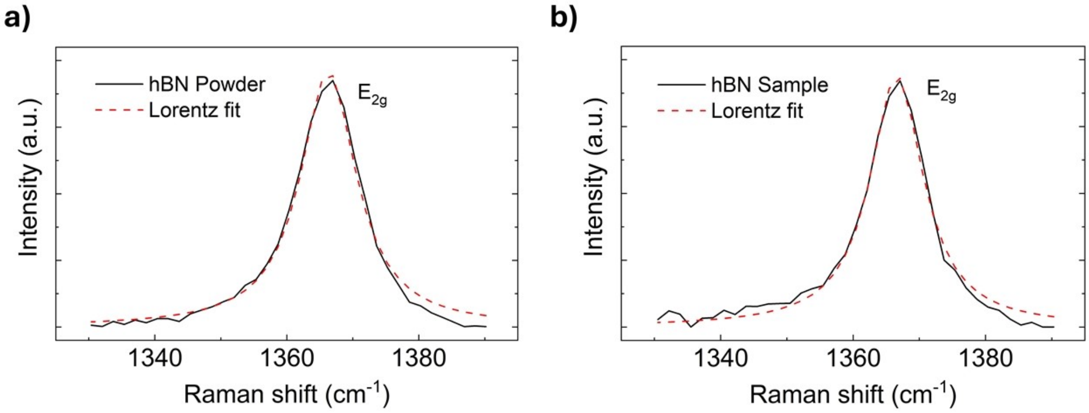


Figure S11 - Raman spectra of the precursor hBN power (left) and the hBN sample (right), both deconvoluted according to a Lorentz peak fit.

Table S6 summarizes the characteristic peaks for the hBN samples.

Table S6 - Raman fit characteristics of the $E_{2g}$ peaks of the hBN sample and its precursor powder.

| hBN | $E_{2g}$ band ($cm^{-1}$) | $E_{2g}$ band FWHM ($\Gamma_G$) |
|---|---|---|
| Powder | 1366.3 | 10.3 cm-1 |
| Sample | 1366.6 | 9.8 cm-1 |

Raman spectra of some $V_2O_5$ samples is shown on Figure S11. Due to the high boiling point of cyrene, its complete removal from substrates requires high processing temperatures which induce the formation of $V_4O_9$ when temperatures above 300 °C are used. This is visible by the appearance of bands in the spectrum at 759 $cm^{-1}$, 907 $cm^{-1}$ (with a shoulder at 892 $cm^{-1}$), and 949 $cm^{-1}$ [39].

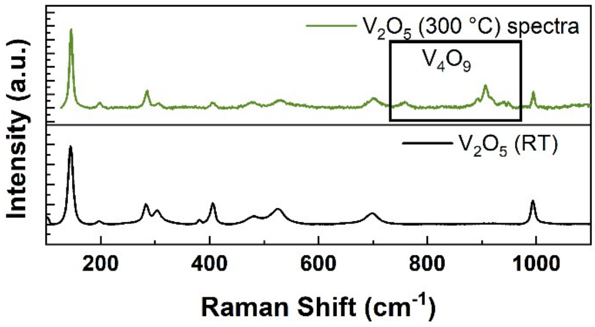


Figure S12 - Comparison between filtered $V_2O_5$ filtered at room temperature bottom spectrum and deposited by spray-coating at 300 °C showing characteristic peaks of $V_4O_9$.

The $B_{2g}$ and $B_{3g}$ peaks of the $MoO_3$ powder and $MoO_3$ sample were determined by fitting the peaks with Lorentz functions, as seen on Figure S13.

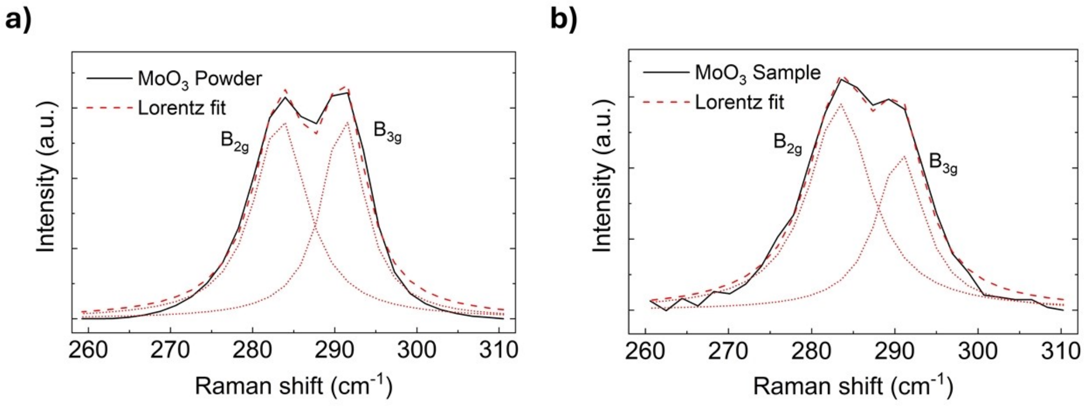


Figure S13 - Raman spectra of the precursor MoO3 power (left) and the MoO3 sample (right), both deconvoluted according to Lorentz peak fits.

The corresponding peak characteristics of the $MoO_3$ powder and $MoO_3$ sample are shown below on Table S7.

Table S7 – Raman peak characteristics of the $MoO_3$ powder and sample.

| $MoO_3$ | $B_{2g}$ band ($cm^{-1}$) | $B_{2g}$ band intensity (a.u.) | $B_{3g}$ band ($cm^{-1}$) | $B_{3g}$ band intensity (a.u.) | $I_{285}/I_{295}$ |
|---|---|---|---|---|---|
| Powder | 283.4 | 142537 | 291.1 | 143524 | 0.99 |
| Sample | 283.4 | 8703 | 290.7 | 6620 | 1.31 |

The spectra of the $MoS_2$ sample using the 532 nm and the 633 nm excitation wavelengths are shown on Figure S14. These result in non-resonant and resonant Raman spectra, respectively. The sample shows the characteristic spectrum of $MoS_2$ for both incident wavelengths [40-42]. The non-resonant spectrum has three main modes: $E_{1g}$ (286 $cm^{-1}$), $E^1_{2g}$ (382 $cm^{-1}$), and $A_{1g}$ (408 $cm^{-1}$). Another weak peak at 450 $cm^{-1}$ corresponds to a 2LA(M) mode. While the $E_{1g}$ (286 $cm^{-1}$) band does not appear in the resonant spectra, both the $E^1_{2g}$ (382 $cm^{-1}$) and $A_{1g}$ (408 $cm^{-1}$) modes are again present. Other contributing modes are also labeled and are described in detail elsewhere [40-42].

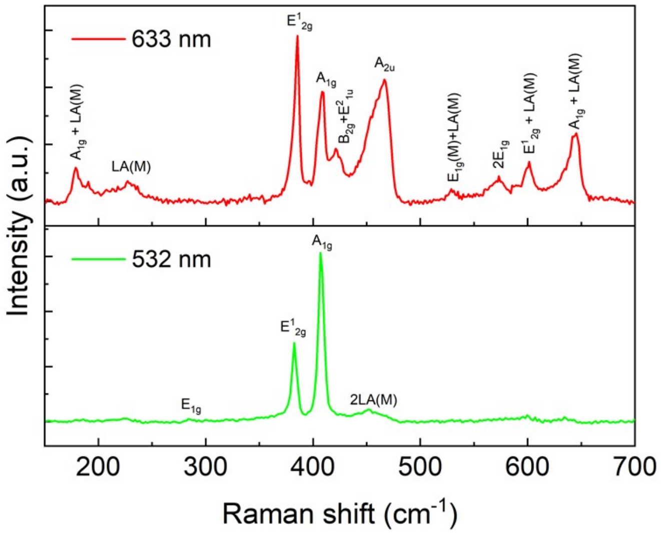


Figure S14 - Non-resonant (top) and resonant (bottom) Raman spectra of the $MoS_2$ sample.

Focusing on the spectra taken with 532 nm in both the $MoS_2$ powder and sample, the $E^1_{2g}$ and $A_{1g}$ peaks were deconvoluted following Lorentzian peak fits as seen on Figure S15 [40, 43].

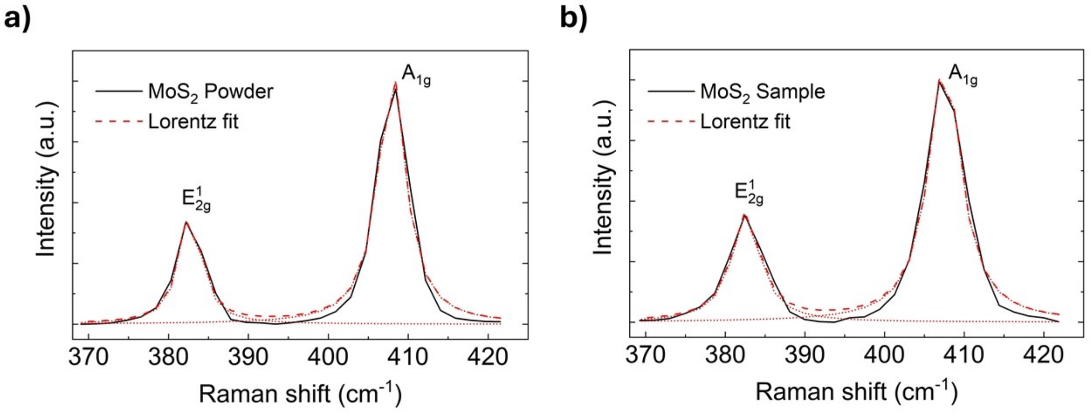


Figure S15- Raman spectra of the precursor $MoS_2$ power (left) and the $MoS_2$ sample (right), both deconvoluted according to Lorentz peak fits.

The corresponding peak characteristics of the $MoS_2$ powder and sample are shown below on Table S8.

Table S8 – Raman peak characteristics of the $MoS_2$ powder and $MoS_2$ sample.

| $MoS_2$ | $E^1_{2g}$ band ($cm^{-1}$) | $E^1_{2g}$ band FWHM ($cm^{-1}$) | $A_{1g}$ band ($cm^{-1}$) | $A_{1g}$ band FWHM ($cm^{-1}$) | $E^1_{2g}$ - $A_{1g}$ ($cm^{-1}$) |
|---|---|---|---|---|---|
| Powder | 382.7 | 3.3 | 408.0 | 4.2 | 25.3 |
| Sample | 382.6 | 4.4 | 407.6 | 4.9 | 25.0 |

# 5. Additional AFM imaging

The following Figure S16 shows AFM used in the determination of flake thickness. Most materials formed large agglomerates, particularly hBN which yielded the highest AFM thickness out of all materials. AFM seems to indicate that all materials formed content of multi-layered stacks with low L:h ratios ranging from 3 to 9.

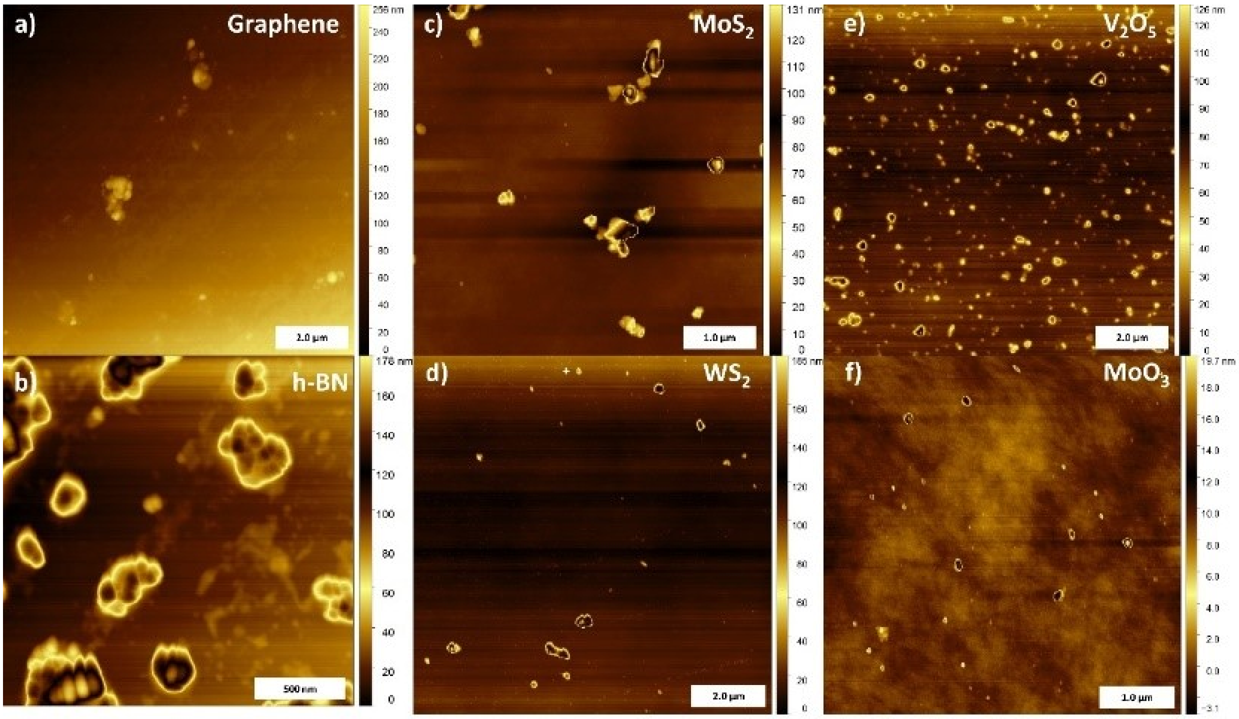


Figure S16 – AFM imaging for samples centrifuged at 6000 rpm and redispersed in IPA: (a) graphene, (b) hBN, (c) $MoS_2$, (d) $WS_2$, (e) $V_2O_5$ and (f) $MoO_3$

# 6. Precursor powder  and exfoliated material SEM imaging

The following Figure S17 and Figure S18 show the precursor flakes and exfoliated materials, respectively.

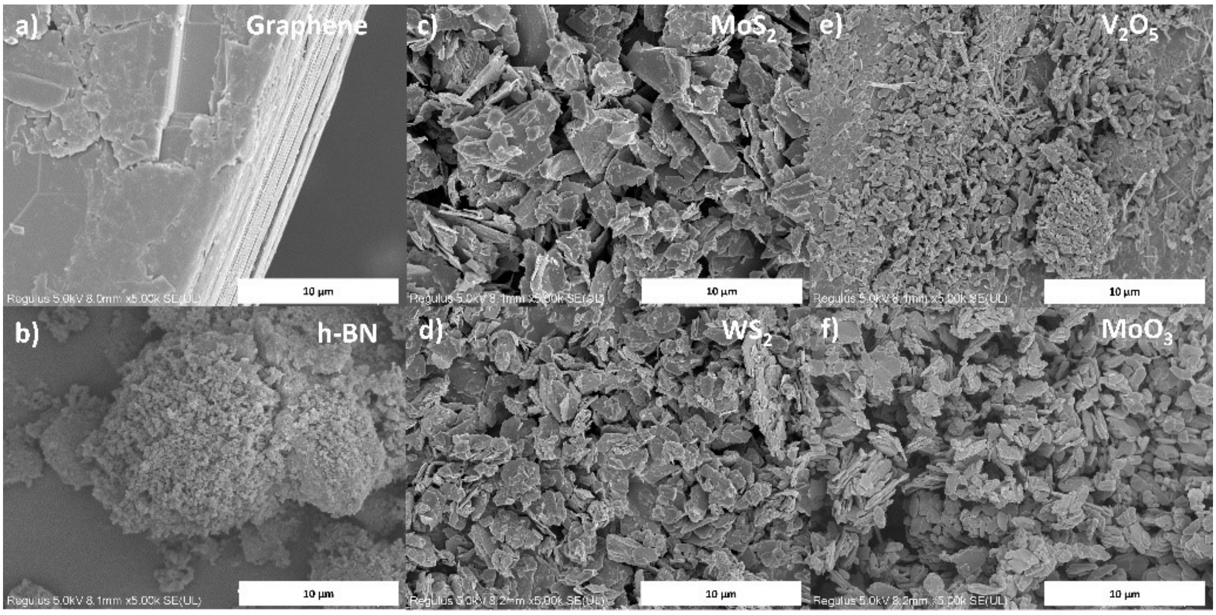


Figure S17 – SEM imaging of unexfoliated powders for (a) graphite, (b) hBN, (c) $MoS_2$, (d) $WS_2$, (e) $V_2O_5$ and (f) $MoO_3$.

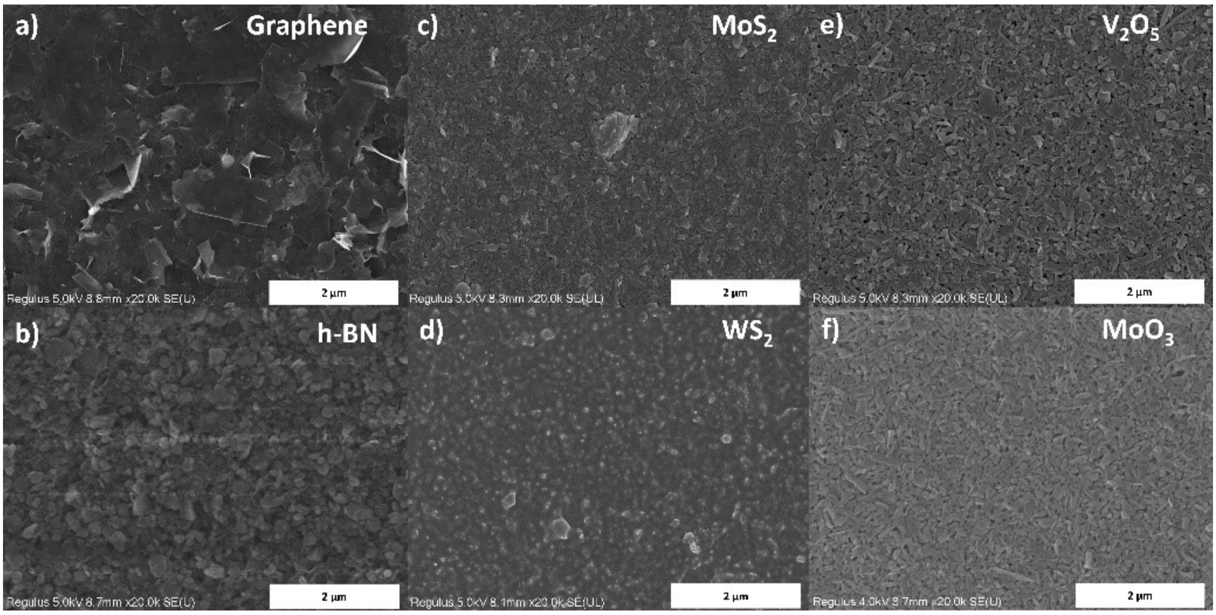


Figure S18 - SEM imaging of 2D materials exfoliated in Cyrene: (a) graphene, (b) hBN, (c) $MoS_2$, (d) $WS_2$, (e) $V_2O_5$ and (f) $MoO_3$.

# 7. Additional HRTEM imaging

# References


1. Y. Hernandez, V. Nicolosi, M. Lotya, F. M. Blighe, Z. Sun, S. De, I. T. McGovern, B. Holland, M. Byrne, Y. K. Gun'Ko, J. J. Boland, P. Niraj, G. Duesberg, S. Krishnamurthy, R. Goodhue, J. Hutchison, V. Scardaci, A. C. Ferrari and J. N. Coleman, *Nat Nanotechnol*, 2008, **3**, 563-568.
2. J. N. Coleman, M. Lotya, A. O'Neill, S. D. Bergin, P. J. King, U. Khan, K. Young, A. Gaucher, S. De, R. J. Smith, I. V. Shvets, S. K. Arora, G. Stanton, H. Y. Kim, K. Lee, G. T. Kim, G. S. Duesberg, T. Hallam, J. J. Boland, J. J. Wang, J. F. Donegan, J. C. Grunlan, G. Moriarty, A. Shmeliov, R. J. Nicholls, J. M. Perkins, E. M. Grieveson, K. Theuwissen, D. W. McComb, P. D. Nellist and V. Nicolosi, *Science*, 2011, **331**, 568-571.
3. B. Radisavljevic, A. Radenovic, J. Brivio, V. Giacometti and A. Kis, *Nat Nanotechnol*, 2011, **6**, 147-150.
4. U. Khan, P. May, A. O'Neill, A. P. Bell, E. Boussac, A. Martin, J. Semple and J. N. Coleman, *Nanoscale*, 2013, **5**, 581-587.
5. R. Durge, R. V. Kshirsagar and P. Tambe, *Procedia Engineering*, 2014, **97**, 1457-1465.
6. D. Hanlon, C. Backes, T. M. Higgins, M. Hughes, A. O'Neill, P. King, N. McEvoy, G. S. Duesberg, B. Mendoza Sanchez, H. Pettersson, V. Nicolosi and J. N. Coleman, *Chemistry of Materials*, 2014, **26**, 1751-1763.
7. J. Zheng, H. Zhang, S. Dong, Y. Liu, C. T. Nai, H. S. Shin, H. Y. Jeong, B. Liu and K. P. Loh, *Nat Commun*, 2014, **5**, 2995.
8. V. Stengl, J. Henych, M. Slusna and P. Ecorchard, *Nanoscale Res Lett*, 2014, **9**, 167.
9. S.-L. Zhang, H.-H. Choi, H.-Y. Yue and W.-C. Yang, *Current Applied Physics*, 2014, **14**, 264-268.
10. Y. Arao and M. Kubouchi, *Carbon*, 2015, **95**, 802-808.
11. P. K. M. K, S. Shanthini and C. Srivastava, *RSC Advances*, 2015, **5**, 53865-53869.
12. X. Fan, P. Xu, D. Zhou, Y. Sun, Y. C. Li, M. A. Nguyen, M. Terrones and T. E. Mallouk, *Nano Lett*, 2015, **15**, 5956-5960.
13. J. Kim, S. Kwon, D. H. Cho, B. Kang, H. Kwon, Y. Kim, S. O. Park, G. Y. Jung, E. Shin, W. G. Kim, H. Lee, G. H. Ryu, M. Choi, T. H. Kim, J. Oh, S. Park, S. K. Kwak, S. W. Yoon, D. Byun, Z. Lee and C. Lee, *Nat Commun*, 2015, **6**, 8294.
14. M. Noroozi, A. Zakaria, S. Radiman and Z. Abdul Wahab, *PLoS One*, 2016, **11**, e0152699.
15. F. Ghasemi and S. Mohajerzadeh, *ACS Appl Mater Interfaces*, 2016, **8**, 31179-31191.
16. R. K. Jha and P. K. Guha, *Nanotechnology*, 2016, **27**, 475503.
17. H. J. Salavagione, J. Sherwood, M. De bruyn, V. L. Budarin, G. J. Ellis, J. H. Clark and P. S. Shuttleworth, *Green Chemistry*, 2017, **19**, 2550-2560.
18. C. Zhang, S.-H. Park, S. E. O'Brien, A. Seral-Ascaso, M. Liang, D. Hanlon, D. Krishnan, A. Crossley, N. McEvoy, J. N. Coleman and V. Nicolosi, *Nano Energy*, 2017, **39**, 151-161.
19. E. T. Bjerglund, M. E. P. Kristensen, S. Stambula, G. A. Botton, S. U. Pedersen and K. Daasbjerg, *ACS Omega*, 2017, **2**, 6492-6499.
20. M. El Garah, S. Bertolazzi, S. Ippolito, M. Eredia, I. Janica, G. Melinte, O. Ersen, G. Marletta, A. Ciesielski and P. Samorì, *FlatChem*, 2018, **9**, 33-39.
21. F. Mori, M. Kubouchi and Y. Arao, *Journal of Materials Science*, 2018, **53**, 12807-12815.
22. J. Peng, Y. Liu, X. Luo, J. Wu, Y. Lin, Y. Guo, J. Zhao, X. Wu, C. Wu and Y. Xie, *Adv Mater*, 2019, **31**, e1900568.
23. C. Backes, D. Campi, B. M. Szydlowska, K. Synnatschke, E. Ojala, F. Rashvand, A. Harvey, A. Griffin, Z. Sofer, N. Marzari, J. N. Coleman and D. D. O'Regan, *ACS Nano*, 2019, **13**, 7050-7061.
24. F. I. Alzakia, B. Tang, S. J. Pennycook and S. C. Tan, *Materials Horizons*, 2020, **7**, 3325-3338.
25. J. Li, J. Han, H. Li, X. Fan and K. Huang, *Materials Science in Semiconductor Processing*, 2020, **107**.
26. Ö. Güler and A. Sönmez, *Journal of Electronic Materials*, 2020, **49**, 5335-5345.

27. Ö. Güler, M. Tekeli, M. Taşkın, S. H. Güler and I. S. Yahia, *Ceramics International*, 2021, **47**, 521-533.
28. L. Houseman, S. Mukherjee, R. Andris, M. J. Zachman and E. Pomerantseva, *Materials Advances*, 2021, **2**, 2711-2718.
29. S. Tkachev, M. Monteiro, J. Santos, E. Placidi, M. B. Hassine, P. Marques, P. Ferreira, P. Alpuim and A. Capasso, *Advanced Functional Materials*, 2021, **31**.
30. A. Roy, P. Kalita and B. Mondal, *Journal of Materials Science: Materials in Electronics*, 2023, **34**.
31. R. Zhang, T. Averianov, R. Andris, M. J. Zachman and E. Pomerantseva, *The Journal of Physical Chemistry C*, 2023, **127**, 919-929.
32. J. Adam, M. Singh, A. Abduvakhidov, M. R. Del Sorbo, C. Feoli, F. Hussain, J. Kaur, A. Mirabella, M. Rossi, A. Sasso, M. Valadan, M. Varra, G. Rusciano and C. Altucci, *Int J Mol Sci*, 2023, **24**.
33. L. Tian, J. Liu, X. Chen, P. S. Branicio and Q. Lei, *Advanced Electronic Materials*, 2024, DOI: 10.1002/aelm.202400143.
34. P. G. Moreira, R. Martins, E. Carlos and R. Branquinho, *Flexible and Printed Electronics*, 2024, **9**.
35. A. C. Ferrari and D. M. Basko, *Nat Nanotechnol*, 2013, **8**, 235-246.
36. Y. Hwangbo, C.-K. Lee, A. E. Mag-Isa, J.-W. Jang, H.-J. Lee, S.-B. Lee, S.-S. Kim and J.-H. Kim, *Carbon*, 2014, **77**, 454-461.
37. V. Kumar, A. Kumar, D. J. Lee and S. S. Park, *Materials (Basel)*, 2021, **14**.
38. S. Roscher, R. Hoffmann and O. Ambacher, *Analytical Methods*, 2019, **11**, 1224-1228.
39. P. Shvets, O. Dikaya, K. Maksimova and A. Goikhman, *Journal of Raman Spectroscopy*, 2019, **50**, 1226-1244.
40. X. Zhang, X. F. Qiao, W. Shi, J. B. Wu, D. S. Jiang and P. H. Tan, *Chem Soc Rev*, 2015, **44**, 2757-2785.
41. M. Ye, D. Winslow, D. Zhang, R. Pandey and Y. Yap, *Photonics*, 2015, **2**, 288-307.
42. H. Li, Q. Zhang, C. C. R. Yap, B. K. Tay, T. H. T. Edwin, A. Olivier and D. Baillargeat, *Advanced Functional Materials*, 2012, **22**, 1385-1390.
43. M. Xie, C. Yun, X. Wang, K. He, B. Liu, J. Zhao, X. Gao, D. Zhang and G. Zhang, *RSC Adv*, 2025, **15**, 7472-7479.
